# Functional characterization of Vip3Aa from *Bacillus thuringiensis* reveals the contributions of specific domains to its insecticidal activity

**DOI:** 10.1016/j.jbc.2023.103000

**Published:** 2023-02-09

**Authors:** Kun Jiang, Zhe Chen, Yuanrong Zang, Yiting Shi, Chengbin Shang, Xuyao Jiao, Jun Cai, Xiang Gao

**Affiliations:** 1 State Key Laboratory of Microbial Technology, Shandong University, Qingdao, China; 2 School of Life Sciences, Shandong University, Qingdao, China; 3 Taishan College, Shandong University, Jinan, China; 4 Department of Microbiology, College of Life Sciences, Nankai University, Tianjin, China

**Keywords:** bacterial pesticidal protein, insecticidal processes, activation mechanism, peritrophic matrix, molecular insecticidal mechanism, BBMV, brush border membrane vesicles, Bt, Bacillus thuringiensis, DI, domain I, DII, domain II, DIII, domain III, DIV, domain IV, DLS, dynamic light scattering, DV, domain V, MJ, midgut juice, MST, microscale thermophoresis, PMa, peritrophic matrix, PTS, Protein thermal shift, Vip3, vegetative insecticidal protein

## Abstract

Microbially derived, protein-based biopesticides offer a more sustainable pest management alternative to synthetic pesticides. Vegetative insecticidal proteins (Vip3), multidomain proteins secreted by *Bacillus thuringiensis*, represent a second-generation insecticidal toxin that has been preliminarily used in transgenic crops. However, the molecular mechanism underlying Vip3’s toxicity is poorly understood. Here, we determine the distinct functions and contributions of the domains of the Vip3Aa protein to its toxicity against *Spodoptera frugiperda* larvae. We demonstrate that Vip3Aa domains II and III (DII-DIII) bind the midgut epithelium, while DI is essential for Vip3Aa’s stability and toxicity inside the protease-enriched host insect midgut. DI-DIII can be activated by midgut proteases and exhibits cytotoxicity similar to full-length Vip3Aa. In addition, we determine that DV can bind the peritrophic matrix *via* its glycan-binding activity, which contributes to Vip3Aa insecticidal activity. In summary, this study provides multiple insights into Vip3Aa’s mode-of-action which should significantly facilitate the clarification of its insecticidal mechanism and its further rational development.

Microbially derived insecticidal proteins are useful substitutes for synthetic pesticides due to their highly targeted insecticidal effects and more sustainable manners (1, 2, 3, 4, 5, 6). *Bacillus thuringiensis* (Bt) is the most broadly used microbial insecticide worldwide due to its production of highly effective Cry insecticidal proteins (7, 8), which account for over 75% of the microbial biopesticide market (9, 10). Cry proteins are produced by Bt during sporulation and are widely used for biological control of pest insects, in the form of transgenic crops and formulated sprays (7, 11, 12, 13, 14). However, the application of Cry proteins also has its limitations due to their singular composition, limited insecticidal spectrum, and increased development of resistance in target pests (1, 2, 8, 9, 15). Therefore, the development of new effective insecticidal protein resources is imperative (3, 4, 7, 9).

Vegetative insecticidal protein (Vip3) family proteins are exotoxins secreted by Bt during its vegetative growth phase (16, 17). Vip3 proteins share no sequence homology with Cry proteins, bind to different receptors, lack cross-resistance, and have efficient broad-spectrum insecticidal activity, especially against lepidopteran pests. These features qualify them as a new generation of insecticidal proteins (3, 16, 18, 19, 20). Additionally, Vip3 proteins have been used in commercial transgenic crops in combination with Cry proteins, exhibiting high insecticidal efficacy, and the development of practical field resistance in target pests has not been reported to date (3, 18), which indicates their promising application potential in crop protection and insecticide resistance management.

Vip3 toxins are multidomain proteins (∼790 amino acids) consisting of a conserved N-terminus and a variable C-terminal region. To date, more than 130 Vip3 proteins have been identified across different Bt strains (21, 22). Since their discovery in 1996 (17), numerous studies have explored the function, structure, and mode of action of Vip3 family proteins. It is known that Vip3 proteins are produced as inactive protoxins. Proteolysis by trypsin or midgut proteases of host insects transforms these inactive protoxins into activated toxins (23, 24, 25). The ∼89 kDa Vip3 proteins are cleaved into two fragments of about 20 kDa (corresponding to the N-terminal ∼198 amino acids) and 66 kDa (corresponding to the C-terminal fragment) (26, 27). After proteolysis, the two fragments were demonstrated to remain tightly associated (28), which is essential for Vip3’s toxicity (29, 30). The toxicity mechanism of Vip3 proteins to the midgut brush border of columnar cells of pest larvae is not yet fully understood. Following binding to host cell surface receptors, pore forming was proposed as the primary toxic mechanism of Vip3 (23, 24), however, apoptosis associated with endocytosis was also observed (19, 31, 32).

Recently, the crystal and cryo-EM structures of Vip3 protoxins- and trypsin-activated Vip3 toxins have been reported successively (33, 34, 35, 36), showing that Vip3 proteins consists of five domains and assemble into highly stable tetramers in both states. After cleavage between domain I (DI) and domain II (DII) by trypsin, Vip3 proteins are shown to undergo a huge conformational change from a tetrameric “pyramid-shaped” protoxin to a tetrameric “syringe-like” activated toxin (34, 36). The N terminus of DI is remodeled into an extended four-helix bundle that can interact with the liposome membrane, which is proposed to be required for pore formation by Vip3 proteins (36). Despite intensive studies investigating their functional mechanism, due to their complicated structural composition and complex biological processes inside the host insect midgut, many aspects of the molecular mode of action of Vip3 proteins—such as the specific relationships between the structures and functions of each domain and the detailed processes of toxicity in the insect midgut—remain unclear, which significantly limits their efficient application in biological pest control.

In this study, we explored the contributions of the various domains of the Vip3Aa to its toxicity in the midgut of *Spodoptera frugiperda* larvae. We found that, together, DII and domain III (DIII) of Vip3Aa can bind to the midgut epithelium. DI-DIII can be activated by midgut proteases and show similar cytotoxicity to activated Vip3Aa toxin. DI is essential for the stability and toxicity of Vip3Aa by maintaining the proper tetramerization of the Vip3Aa protoxin. Furthermore, domain V (DV) binds to the peritrophic matrix (PMa) *via* its glycan-binding ability, which contributes to the insecticidal activity of Vip3Aa. Together, our study reveals the various domains function of Vip3Aa and provides multiple insights into its molecular mode of action.

## Results

### Vip3Aa binds to the midgut epithelium of *S. frugiperda* larvae through domain II and domain III

**Figure 1:**
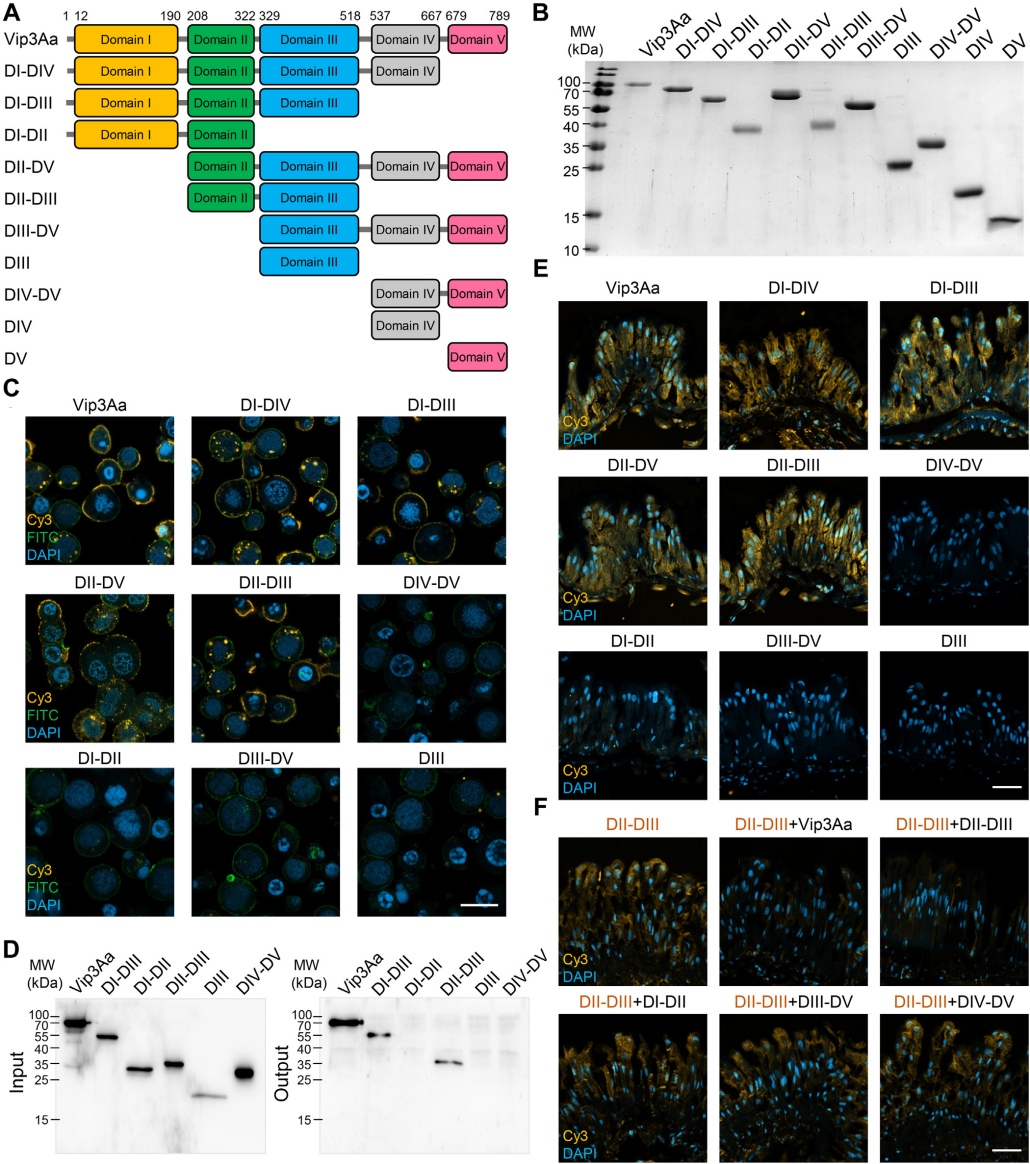
**Vip3Aa specifically binds to host midgut epithelium *via* DII and DIII.***A*, a schematic diagram of Vip3Aa and its truncation variants assessed with fluorescence staining. *B*, SDS-PAGE analysis of the Cy3-labeled Vip3Aa and the Vip3Aa truncation variants listed in *A*. *C*, confocal microscopy of Sf9 cells treated with Cy3 fluorescence-labeled Vip3Aa or its truncation variants *(yellow)*. Nuclei are stained with DAPI (*blue*), and cell membranes are stained with FITC-phalloidin (*green*). The scale bar represents 20 μm. *D*, Western blotting to analyze the binding of Vip3Aa and its truncation variants to *Spodoptera frugiperda* BBMV. Shown is a representative of three independent experiments. *E*, confocal microscopy images showing binding of Cy3 fluorescence-labeled Vip3Aa and its truncation variants *(yellow)* to the midgut tissue of *S. frugiperda* larva. Nuclei are stained with DAPI *(blue)*. The scale bar represents 50 μm. *F*, confocal microscopy images showing the effect of an excess of unlabeled (40 μM) Vip3Aa, DII-DIII, DI-DII, DIII-DV, and DIV-DV on the binding of Cy3-labeled DII-III (0.1 μM) *(yellow)* to the midgut epithelium. Nuclei are stained with DAPI *(blue)*. The scale bar represents 50 μm. In *C*, *E*, and *F*, the images represent at least 30 images from three independent experiments. BBMV, brush border membrane vesicles; DAPI, 4′,6-diamidino-2-phenylindole; DI, domain I; DII, domain II; DIII, domain III; DIV, domain IV; DV, domain V; Vip3, vegetative insecticidal protein.

Intensive studies have shown that the multidomain Vip3 proteins can bind to Sf9 cells, Sf21 cells, and brush border membrane vesicles (BBMV) of the insect midgut, and this binding has been shown to confer cytotoxic effects (19, 20, 28, 37, 38). However, it is still unclear how this family of proteins specifically binds to the midgut epithelium of insect hosts. We constructed and purified full-length Vip3Aa and several truncation variants based on its domain composition (34, 35) (Fig. 1*A*); we then fluorescently labeled them with Cy3 dye (Fig. 1*B*). First, we determined their ability to bind to commercially available Sf9 cells derived from *S. frugiperda* ovaries. Fluorescence microscopy showed that the binding capability of DI-Domain IV (DI-DIV), DI-DIII, DII-DV, and DII-DIII to Sf9 cells resembled that of the full-length Vip3Aa protein. However, DIV-DV, DI-DII, DIII-DV, and DIII did not bind to Sf9 cells (Fig. 1*C*), indicating that only DII and DIII together are required for binding to Sf9 cells. Consistent with the results for Sf9 cell binding, the binding analysis to *S. frugiperda* BBMV also revealed that DII-DIII can bind to the BBMV as DI-DIII and Vip3Aa, while DIV-DV cannot (Fig. 1*D*).

We next investigated the binding capability of Vip3Aa to the midgut of *S. frugiperda* larvae. Specifically, we prepared frozen epithelial tissue sections from *S. frugiperda* midgut and incubated them with Cy3-labeled Vip3Aa and truncation variants. Consistent with the results for Sf9 cell and BBMV binding, we found that both DII and DIII are required for Vip3Aa binding to the midgut epithelium (Fig. 1*E*). To confirm the specific requirement of DII-DIII for binding of the midgut epithelium, we conducted competitive binding assays. Fluorescence microscopy showed that the addition of unlabeled Vip3Aa or DII-DIII resulted in a significant reduction in the amount of Cy3-labeled DII-DIII bound to the midgut epithelium. By contrast, the addition of unlabeled DI-DII, DIII-DV, or DIV-DV did not affect the level of Cy3-labeled DII-DIII bound to the midgut epithelium (Fig. 1*F*). These results demonstrate that Vip3Aa directly binds to the *S. frugiperda* midgut epithelium *via* DII-DIII.

### Vip3Aa’s cytotoxic effects are specifically mediated by domain I to domain III

Having found that Vip3Aa directly binds to the *S. frugiperda* midgut epithelium through DII-DIII, we next examined post binding activities by testing which specific Vip3Aa domain(s) are responsible for causing damage to the midgut epithelium. Previous studies have established that Vip3 proteins are synthesized as an inactive protoxin and are subsequently proteolytically processed by trypsin or midgut proteases at the loop region between DI and DII (34). This proteolytic processing generates two fragments of around 20 kDa (comprising DI) and 66 kDa (comprising the remainder of the Vip3 proteins) which remain closely associated; these fragments undergo substantial conformational changes that are understood to be essential to their activation and cytotoxicity (26, 29, 34, 35). Therefore, before testing which domain(s) mediate Vip3Aa’s cytotoxic effects against the *S. frugiperda* midgut epithelium, all truncation variants of Vip3Aa capable of binding the midgut epithelium were assessed with proteolysis activation tests, using both trypsin and midgut juice (MJ) from *S. frugiperda*.

**Figure 2:**
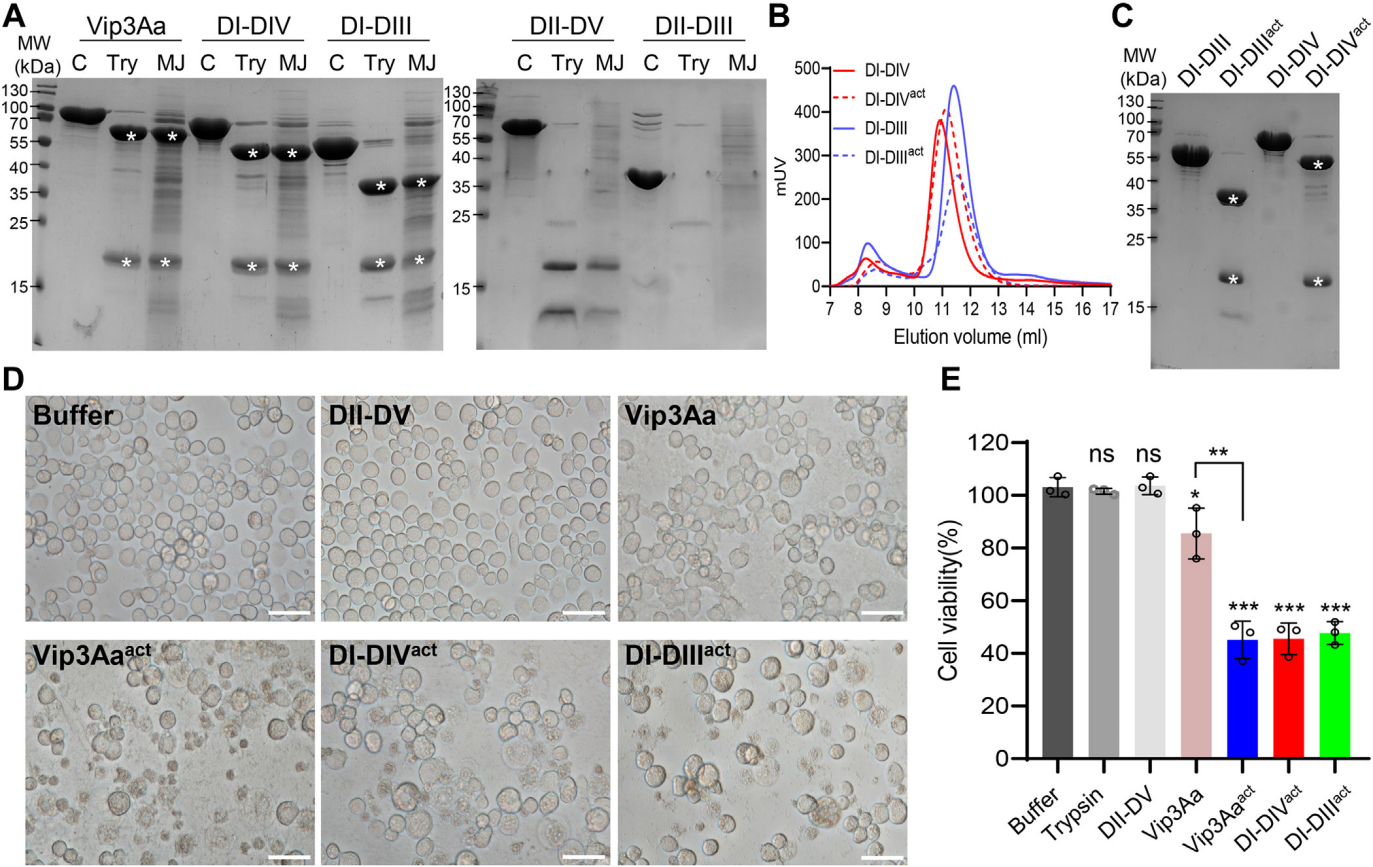
**Domain I–Domain III is responsible for the cytotoxic effects of Vip3Aa.***A*, SDS-PAGE analysis of Vip3Aa, DI-DIV, DI-DIII, DII-DV, and DII-DIII after treatment with trypsin at a ratio of 1: 50 (trypsin:Vip3Aa, wt:wt) or *Spodoptera frugiperda* midgut juice (MJ) at a ratio of 1: 8 (MJ: Vip3Aa, wt:wt). Proteolysis was stopped with the addition of an irreversible protease inhibitor (AEBSF). *Asterisks* indicate the main bands following proteolysis. *B*, size-exclusion chromatography (SEC) analysis of the trypsin-treated DI-DIV (DI-DIV^act^) and DI-DIII (DI-DIII^act^); untreated DI-DIV and DI-DIII were used as controls. The samples were loaded on a Superdex 200 Increase 10/300 Gl column. *C*, SDS-PAGE analysis of the collected peak fractions of DI-DIV, DI-DIV^act^, DI-DIII, and DI-DIII^act^ from the SEC analysis in *B*. *Asterisks* indicate the main bands following proteolysis. *D*, microscopic views of Sf9 cells treated with Vip3Aa, DII-DV, trypsin-activated Vip3Aa (Vip3Aa^act^), DI-DIV^act^, and DI-DIII^act^ for 72 h. The scale bar represents 20 μm. The images represent 30 images from at least three independent experiments. *E*, cell viability of the Sf9 cells from panel *D* and Fig. S1*B*. Data are expressed as the mean ± SD from three independent experiments. Statistical analysis was performed using one-way ANOVA with Duncan’s MRT; ns, nonsignificant; ∗*p* < 0.05; ∗∗*p* < 0.01; ∗∗∗*p* < 0.001. DII, domain II; DIV, domain IV; DV, domain V; Vip3, vegetative insecticidal protein.

SDS-PAGE analysis showed that after trypsin or MJ processing, DI-DIV and DI-DIII still demonstrated consistent proteolytic patterns similar to Vip3Aa, appearing as two major bands corresponding to the 20 kDa (DI) fragment and the remainder of the protein (Fig. 2*A*). In contrast, DII-DV and DII-DIII were almost completely digested and did not produce intense bands greater than 20 kDa, indicating the reduced stability of these two truncation variants (Fig. 2*A*). Additionally, size-exclusion chromatography evaluation and SDS-PAGE analysis further confirmed that the two fragments of DI-DIV or DI-DIII remained tightly associated after proteolytic processing in solution (Fig. 2, *B* and *C*). Therefore, we only examined the toxicity of DI-DIV and DI-DIII of Vip3Aa. Purified trypsin-activated Vip3Aa (act-Vip3Aa), DI-DIV, DI-DIII, and other control proteins were applied to Sf9 cells. Consistent with the previous study, the toxicity of activated Vip3Aa to Sf9 cells was significantly more potent than that of the Vip3Aa protoxin (Fig. 2, *D* and *E*) (37). The cytotoxicity assay further showed that activated DI-DIV, DI-DIII, and Vip3Aa proteins exhibited similar toxicity to Sf9 cells (Figs. 2, *D* and *E*, and S1, *A* and *B*). Our cytotoxicity results were consistent with the conclusions reported by Quan *et al.* (37), who recently showed that DI-III and DI-IV were as toxic to Sf21 cells as the activated full-length Vip3Af. These results together confirmed that the truncated DI-DIII has similar cytotoxicity to the full-length protein, which might be applied to all Vip3A proteins.

### Domain I is essential for the stability and toxicity of Vip3Aa by maintaining the properly folded tetramer of the Vip3Aa protoxin

**Figure 3:**
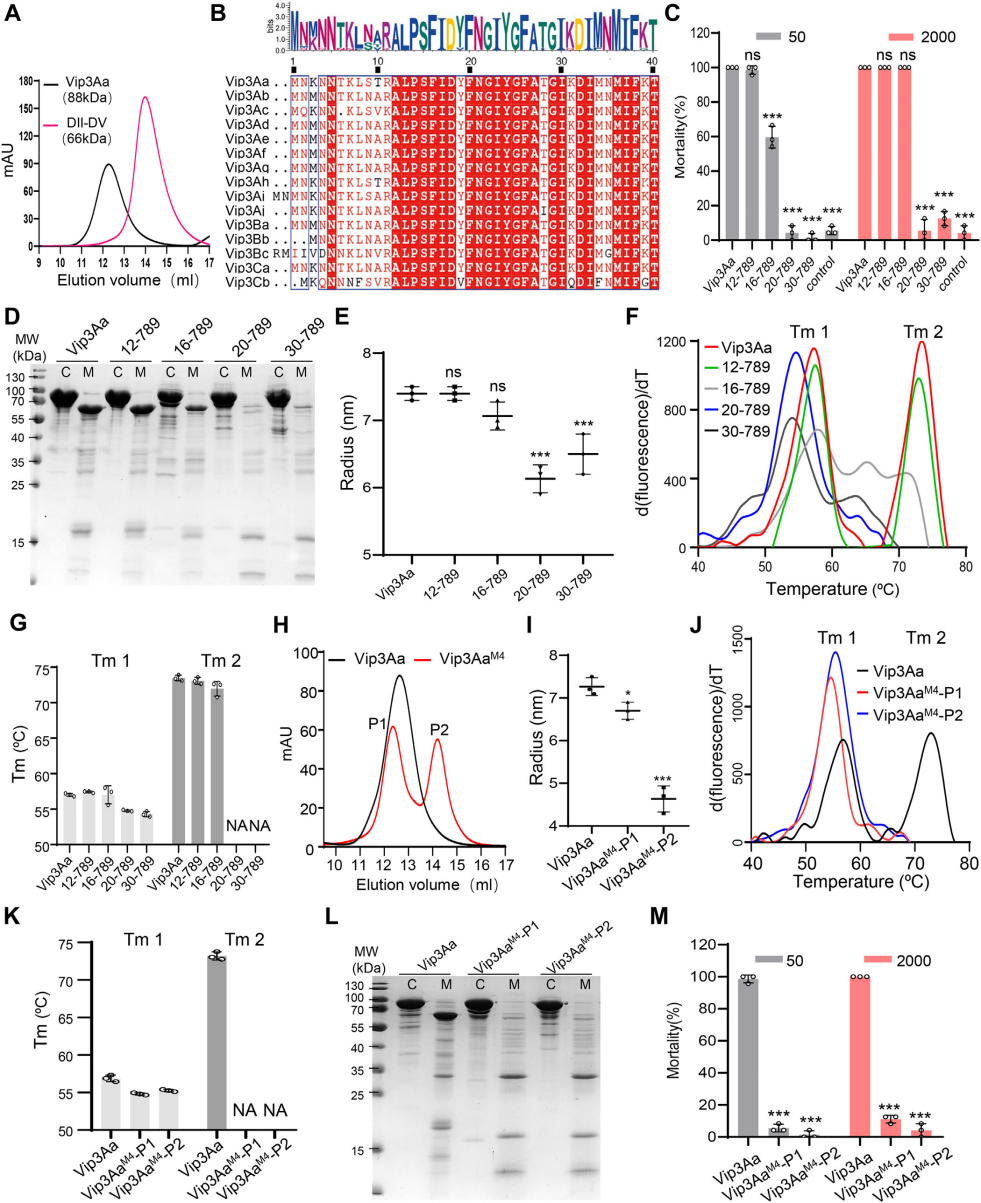
**Domain I is essential for the proper tetramerization of Vip3Aa protoxin and further impacts its stability in the midgut.***A*, size-exclusion chromatography analysis of the purified Vip3Aa and DII-DV. The samples were loaded on a Superdex 200 Increase 10/300 Gl column. *B*, sequence alignment of the N-terminal amino acids of different subclasses of Vip3 family proteins. The Weblogo above was generated using WebLogo 3: Public Beta. *C* and *M*, mortality analysis of *Spodoptera frugiperda* larvae as induced by Vip3Aa and the indicated Vip3Aa mutants at concentrations of 50 and 2000 ng/cm^2^ (n = 24). Data are expressed as the mean ±SD from three independent experiments. Statistical analysis was performed using one-way ANOVA with Duncan’s MRT; ns, nonsignificant; ∗∗∗*p* < 0.001. *D* and *L*, SDS-PAGE analysis of the Vip3Aa and the indicated Vip3Aa mutants after treatment with *S. frugiperda* MJ at a ratio of 16: 1 (Vip3Aa: MJ, wt: wt) at 27 °C for 12 h. “*C*”: the proteins untreated as control; “*M*”: the proteins treated with MJ. The images shown are from at least three independent experiments. *E* and *I*, dynamic light scattering analysis of Vip3Aa and the indicated Vip3Aa mutants (0.5 mg/ml). Data are expressed as the mean ± SD from three independent experiments. Statistical analysis was performed using one-way ANOVA with Duncan’s MRT; ns, nonsignificant; ∗*p* < 0.05; ∗∗∗*p* < 0.001. *F* and *J*, protein thermal shift (PTS) assay analysis of Vip3Aa and the indicated Vip3Aa mutants (0.5 mg/ml). The thermal shift assays curves are representative of three independent repetitions of each sample. *G* and *K*, histograms showing melting temperature values of Vip3Aa and the indicated Vip3Aa mutants in the PTS assays of *F* and *J*. Tm1 and Tm2 respectively indicate the Tm values of peak 1 and peak 2 in the thermal shift assay curves. NA: not available. *H*, size-exclusion chromatography analysis of the purified Vip3Aa and Vip3Aa^M4^. The samples were loaded on a Superdex 200 Increase 10/300 Gl column. P1 and P2 indicate the two peaks of the elution fractions of Vip3Aa^M4^. MJ, midgut juice; Vip3, vegetative insecticidal protein.

DII-DV is highly sensitive to MJ and is almost entirely degraded by it (Fig. 2*A*), which supports previous observations that purified DII-DV of Vip3Aa has no insecticidal activity (35). Structural studies have revealed that both the Vip3 protoxin and trypsin-activated Vip3 toxin assemble into a stable tetramer, and this oligomerization is required for Vip3’s toxicity (29, 30, 34). Through structural analysis, DI has been found to be essential for maintaining the tetramerization of the Vip3Aa protoxin (34). Consistent with this observation, size-exclusion chromatography and dynamic light scattering (DLS) assays showed that, unlike the full-length Vip3Aa protoxin, DII-DV could not form a tetramer (Figs. 3*A* and S2*A*). Therefore, we hypothesized that DI of Vip3Aa functions in maintaining the tetramerization of the Vip3Aa protoxin, which is required for its stability and toxicity.

Through sequence alignment, we found that the sequence identity of DI is relatively high across the Vip3 protein family, excluding the first approximately ten amino acids of Vip3 proteins (Figs. 3*B* and S3), which are speculated to be a potential signal sequence (29, 39). Further structural analysis of the Vip3Aa protoxin revealed that residues 14 to 21 from the DI N-terminus are nested into the groove formed by DI and DIII, forming numerous hydrophobic interactions (Fig. S2, *B* and *C*). We subsequently constructed Vip3Aa_12–789_, Vip3Aa_16–789_, Vip3Aa_20–789_, and Vip3Aa_30–789_ truncation variants (Fig. S2*D*) and evaluated their toxicity against *S. frugiperda* larvae. Unlike the Vip3Aa and Vip3Aa_12–789_ proteins, both of which exhibited clear toxicity against *S. frugiperda* larvae, the toxicity of the Vip3Aa_16–789_ protein was significantly reduced and no toxicity was detected for Vip3Aa_20–789_ and Vip3Aa_30–789_ (Fig. 3*C* and Table 1).

**Table 1:** Mortality analysis of *Spodoptera frugiperda* larvae (first instar) as induced by Vip3Aa and the indicated mutant proteins

Protein	LC50 (ng/cm^2^)	95% Fiducial limit (ng/cm^2^)-lower	95% Fiducial limit (ng/cm^2^)-upper
Vip3Aa	19.32	17.57	21.26
Vip3Aa_12–789_	23.97	21.25	26.75
Vip3Aa_16–789_	44.07	41.33	47.01
Vip3Aa_20–789_	No^a^	N/A	N/A
Vip3Aa_30–789_	No^a^	N/A	N/A
Vip3Aa^M4^	No^a^	N/A	N/A
DI-DIII	No^a^	N/A	N/A
DI-DIV	No^a^	N/A	N/A
Vip3Aa^R704A/K708A^	165.4	149.6	184.0
Vip3Aa^N730A/R732A/R734A^	50.67	44.28	57.28
Vip3Aa^E742A/R744A^	87.82	79.62	97.02
Vip3Aa^Y766A/E768A/S770A^	32.74	28.36	37.21

Abbreviations: DI, domain I; DIII, domain III, DIV, domain IV; DV, domain V; N/A, not applicable; Vip3, vegetative insecticidal protein.

a No: nontoxic.

To determine the contribution of the amino acid residues 12 to 30 to Vip3Aa’s insecticidal activity, we first performed a proteolysis assay on these Vip3Aa truncation variants with MJ from *S. frugiperda*. SDS-PAGE analysis showed that compared to Vip3Aa, Vip3Aa_16–789_ was less stable and the Vip3Aa_20–789_ and Vip3Aa_30–789_ truncation variants were almost completely digested (Fig. 3*D*), which supports the losing insecticidal activity of these Vip3Aa truncation variants. In addition, DLS assays revealed that the particle sizes of Vip3Aa_20–789_ and Vip3Aa_30–789_ were significantly different from that of Vip3Aa (Fig. 3*E*), which suggested that their oligomerization states were distinct from that of Vip3Aa. To further confirm their change in oligomerization states, we performed protein thermal shift (PTS) assays. Vip3Aa shows two thermal transitions (peaks) in the PTS assay, which are close to the peaks corresponding to DIV-DV (peak 1) and DI-DIII (peak 2) (Figs. 3*F* and S4, *A* and *B*). This suggests that peak 2 is produced by the unfolding of tetramerized DI-DIII and peak 1 mainly results from unfolding of DIV-DV. Furthermore, the peak close to peak 2 disappeared in Vip3Aa_20–789_ and Vip3Aa_30–789_ (Fig. 3, *F* and *G*), indicating that these truncation variants could not form properly folded tetramers compared with Vip3Aa, which further suggests that improper tetramerization significantly affected the stability of these variants in the presence of midgut proteases from insect hosts.

To further validate the necessity of proper tetramerization of the Vip3Aa protoxin to its stability and toxicity, we generated a Vip3Aa^M4^ mutant (Vip3Aa^M4^, R175A, K177A, E181A, and K182A). These amino acid residues from DI were shown to be involved in interactions between Vip3Aa monomers and likely facilitate the tetrameric formation of the Vip3Aa protoxin (Fig. S2, *E* and *F*). Vip3Aa^M4^ produced two peaks in size-exclusion chromatography (Fig. 3*H*), which indicates that it has two oligomerization states after purification, one is close to the size of tetramer (P1) and the other is close to the size of monomer (P2). DLS assays showed that the particle sizes of Vip3Aa^M4^ in both oligomerization states (P1 and P2) were significantly smaller than that of WT Vip3Aa (Fig. 3*I*), which suggested that their oligomerization states were distinct from that of Vip3Aa. PTS assays further confirmed that Vip3Aa^M4^ does not form a properly folded tetramer (Fig. 3, *J* and *K*), due to lack of the thermal transition peak (Tm 2) of tetramer. In addition, compared with Vip3Aa, Vip3Aa^M4^ is almost entirely digested by *S. frugiperda* larvae MJ (Fig. 3*L*) and loses its insecticidal activity (Fig. 3*M* and Table 1), which further indicated that this region of DI is important for the proper tetramer formation of Vip3Aa protoxin and proper tetramerization is necessary for Vip3Aa’s stability and toxicity. Together, these results indicate that DI is essential for the stability and toxicity of Vip3Aa in the protease-enriched host insect midgut by maintaining the proper tetramerization of the Vip3Aa protoxin.

### Vip3Aa binds to the PMa of *S. frugiperda* larvae *via* domain V

**Figure 4:**
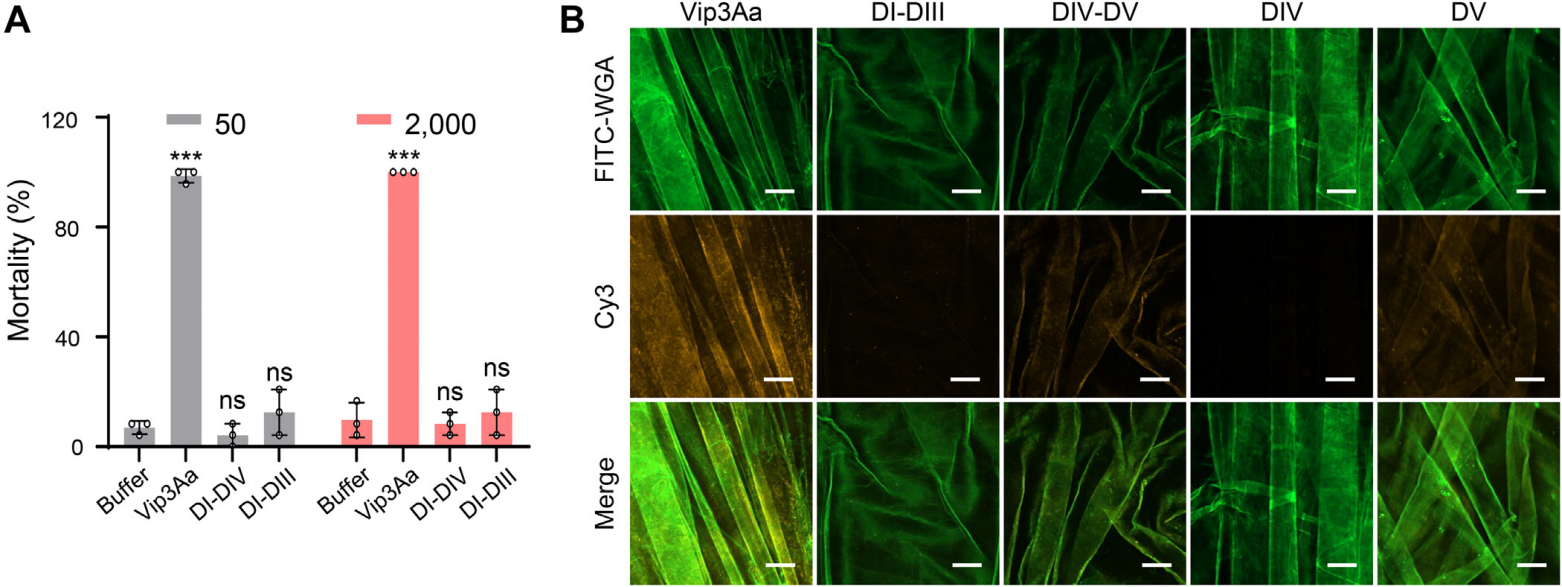
**Vip3Aa binds to the peritrophic matrix of *Spodoptera frugiperda* larvae *via*domain V.***A*, mortality analysis of *S. frugiperda* larvae as induced by Vip3Aa, DI-DIV, and DI-DIII at concentrations of 50 and 2000 ng/cm^2^ (n = 24). Data are expressed as the mean ±SD from three independent experiments. Statistical analysis was performed using one-way ANOVA with Duncan’s MRT; ns, nonsignificant; ∗∗∗*p* < 0.001. *B*, confocal microscopy images showing the binding of Cy3 fluorescence-labeled Vip3Aa and the indicated Vip3Aa mutants *(yellow)* to the PMa of *S. frugiperda* larvae. The PMa is stained with FITC-conjugated wheat germ agglutinin (WGA) *(green)*. The scale bar represents 50 μm. The images represent at least three independent experiments. DI, domain I; DIII, domain III; DIV, domain IV; PMa, peritrophic matrix; Vip3, vegetative insecticidal protein.

As discussed, our results together with the results of Quan *et al.* (37) confirmed that the truncated DI-DIII exerts similar cytotoxicity to full-length Vip3A (Fig. 2, *D* and *E*). However, bioassays showed that neither DI-DIII nor DI-DIV exerted any toxicity against *S. frugiperda* larvae (Fig. 4*A*). This finding indicates that at least DV is required for the insecticidal activity of Vip3Aa inside the insect midgut, a more complex physiological environment. Several structural studies have revealed that DV contains a conserved glycan-binding motif, which may facilitate recognition of glycosylated receptors by Vip3 proteins in the midgut membranes of susceptible insects (33, 34, 35). However, our findings indicate that DV is not involved in binding of the host midgut epithelium (Fig. 1*E*). It has been reported that the PMa—a highly glycosylated layer lining the invertebrate midgut, which is mainly produced by the midgut epithelium or cardia—can be bound by Cry toxins (40, 41). We therefore hypothesized that DV of Vip3Aa might bind to the PMa. Pursuing this, we extracted the PMa from *S. frugiperda* larvae (Fig. S5), then used laser confocal microscopy to evaluate the potential binding of Cy3-labeled Vip3Aa protein and truncation variants to the PMa. Vip3Aa, DIV-DV, and DV could bind the PMa, whereas no binding was detected for DI-DIII or DIV (Fig. 4*B*). These findings support that Vip3Aa binds to the PMa *via* DV.

### Binding of the PMa *via* the glycan-binding ability of domain V contributes to Vip3Aa’s insecticidal activity

**Figure 5:**
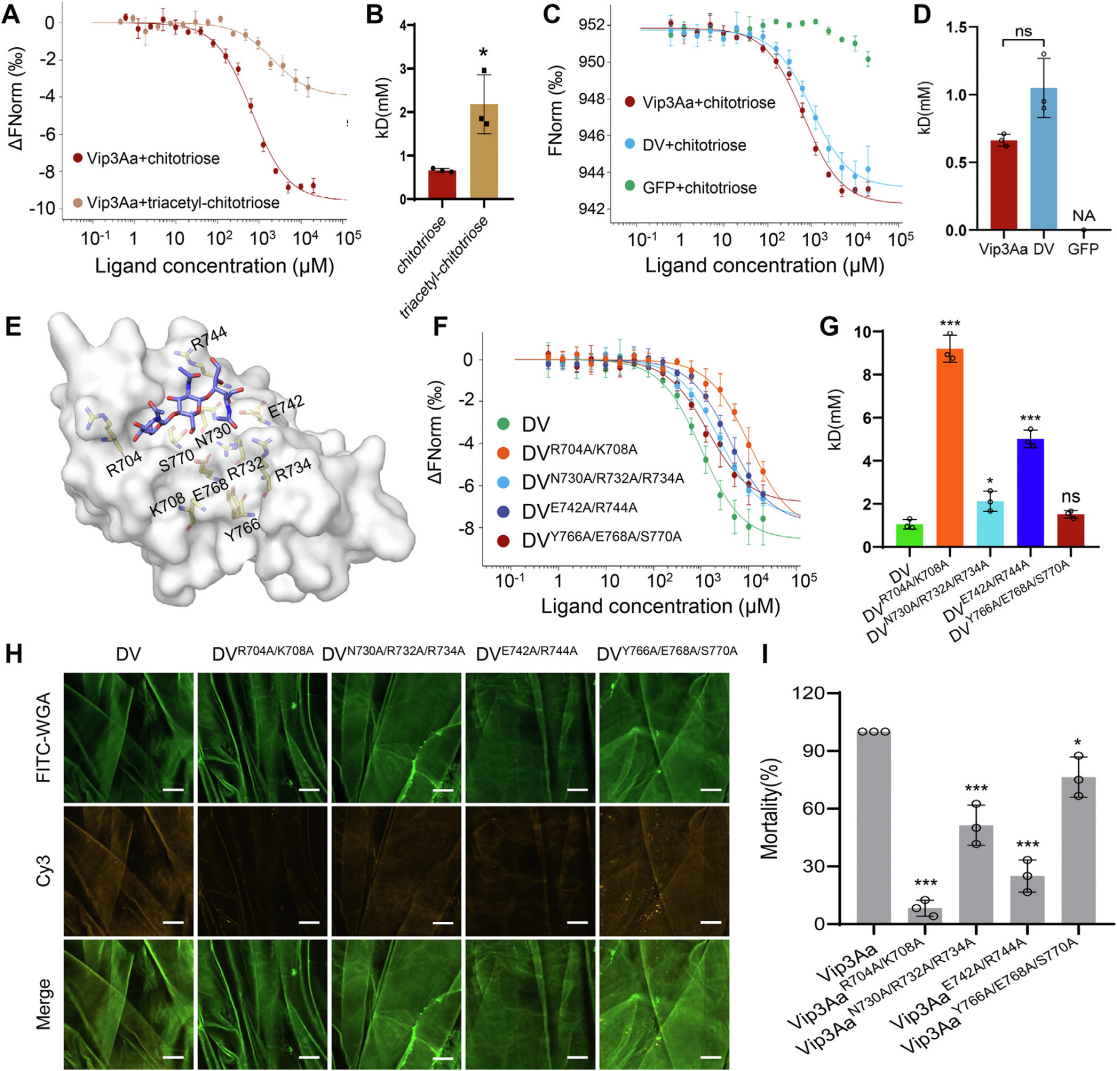
**DomainV binding to the peritrophic matrix through its glycan-binding capability is required for the insecticidal activity of Vip3Aa.***A*, microscale thermophoresis (MST) assay to measure the binding affinities of Vip3Aa with triacetyl chitotriose and chitotriose. *Fitted binding curves* were derived from three independent experiments. *B*, histogram showing the binding affinity Vip3Aa with triacetyl chitotriose and chitotriose measured by the MST assay in *A*. Data are expressed as the mean ±SD from three independent experiments. ∗*p* < 0.05 by unpaired two-tailed Student’s *t*-tests. *C* and *F*, microscale thermophoresis assay to measure the binding affinities of Vip3Aa, DV, and the indicated DV mutants with chitotriose. Fitted binding curves were derived from three independent experiments. *D* and *G*, histograms showing the binding affinity of Vip3Aa, DV, and the indicated DV mutants with chitotriose measured by the MST assays of *C* and *F*. Data are expressed as the mean ±SD from three independent experiments; ns, nonsignificant; ∗*p* < 0.05; ∗∗∗*p* < 0.001; unpaired two-tailed Student’s *t*-tests in *D*; one-way ANOVA using Duncan’s MRT in *G*. *E*, molecular modeling of chitotriose within the glycan-binding pocket of Vip3Aa DV (PDB: 6vls). The potential amino acid residues engaged in the interactions between DV and chitotriose are shown as *sticks*. Chitotriose is shown as a *stick (in purple)*. *H*, confocal microscopy images showing the binding of Cy3 fluorescence-labeled Vip3Aa and the indicated Vip3Aa mutants *(yellow)* to the PMa of *Spodoptera frugiperda* larvae. The PMa is stained with FITC-conjugated wheat germ agglutinin (WGA) *(green)*. The scale bar represents 50 μm. The images represent at least three independent experiments. *I*, insecticidal activity of Vip3Aa and the indicated Vip3Aa mutants to *S. frugiperda* larvae at a concentration of 50 ng/cm^2^ (n = 24). Data are expressed as the mean ± SD from three independent experiments. Statistical analysis was performed using one-way ANOVA with Duncan’s MRT; ∗*p* < 0.05; ∗∗∗*p* < 0.001. DV, domain V; PMa, peritrophic matrix; Vip3, vegetative insecticidal protein.

We subsequently evaluated whether DV’s glycan-binding ability mediates the observed DV-PMa binding. For context, the chitin net of the PMa is composed of linear polysaccharide polymers comprised of GlcNAc monomers and its derivative D-glucosamine, connected by β-1,4-glycoside bonds, which provide attachment sites for proteins and glycoproteins in the midguts of larvae (42, 43). We initially assayed the binding ability of Vip3Aa to N,N',N''-triacetyl chitotriose (GlcNAc(β1-4)GlcNAc(β1-4)GlcNAc) and chitotriose (GlcNH_2_(β1-4)GlcNH_2_(β1-4)GlcNH_2_). Microscale thermophoresis (MST)-binding assays showed that Vip3Aa could bind to both chitotriose and N, N′, N''-triacetyl chitotriose, and that chitotriose exhibited higher binding affinity to Vip3Aa (Fig. 5, *A* and *B*). Consistent with the glycan-binding capability of Vip3Aa, DV alone could also bind to chitotriose (Fig. 5, *C* and *D*).

Initially, cocrystallization and soaking methods were used in an attempt to obtain the structure of DV in complex with chitotriose, however, neither method was successful. Thus, we adopted a molecular docking approach to investigate the protein–ligand interactions. A chitotriose-bound DV structural model identified several amino acid residues positioned around the chitotriose that are likely involved in the binding activity (Figs. 5*E* and S6*A*). To examine the potential functional significance of the observed chitotriose-binding amino acid residues, we introduced structurally guided mutations into DV residues (R704A/K708A, N730A/R732A/R734A, E742A/R744A, and Y766A/E768A/S770A) (Fig. S6*B*) and evaluated their binding ability to bind chitotriose. MST assays showed that the binding affinity of three of the four variants to chitotriose was less than that of DV, especially DV^R704A/K708A^ and DV^E742A/R744A^ (Fig. 5, *F* and *G*). In accordance with the glycan-binding results above, laser confocal microscopy showed that binding of DV^R704A/K708A^ and DV^E742A/R744A^ to the PMa was clearly reduced compared to unmodified DV (Fig. 5*H*). Additionally, the addition of chitotriose to the sample mixtures significantly attenuated the binding of DV to the PMa (Fig. S6*C*), providing additional support that DV-PMa binding is mediated by DV’s glycan-binding ability.

To assess whether reducing the extent of DV binding to the PMa reduces the toxicity of Vip3Aa, we conducted bioassays in which *S. frugiperda* larvae were treated with Vip3Aa mutant variants (Vip3Aa^R704A/K708A^, Vip3Aa^N730A/R732A/R734A^, Vip3Aa^E742A/R744A^, and Vip3Aa^Y766A/E768A/S770A^) (Fig. S7, *A* and *B*). Consistent with our prior observations, the toxicity of Vip3Aa^R704A/K708A^ and Vip3Aa^E742A/R744A^ was significantly reduced compared with Vip3Aa by about 8 and 4.5 times, respectively (Figs. 5*I*, S7*C* and Table 1). Together, these results show that DV directly binds to the PMa of *S. frugiperda* larvae through its glycan-binding ability and demonstrate that this binding contributes to the insecticidal activity of Vip3Aa.

## Discussion

Using entomopathogens to manage various detrimental insect pests is an important sustainable biological control alternative to using synthetic pesticides (5, 6). Currently, about 16 classes of bacterial pesticidal proteins have been identified (21, 22). However, only Cry proteins have been extensively studied and successfully used worldwide (7, 8, 11, 12, 13). Vip3 proteins have characteristics and mode-of-action mechanisms distinct from Cry proteins and show high insecticidal activity against lepidopteran pests and are classified as a second generation of insecticidal proteins that can be broadly applied after Cry proteins in the field (3, 16, 18, 19, 44). However, the lack of clear understanding of the molecular mechanisms underlying their insecticidal activity severely restricts their broader application and rational development. Here, we determined the functions of the multiple domains of the Vip3Aa protein. Since all members of the Vip3 family are highly conserved, these results and unique insights can likely be applied to the other members of this protein family.

DI of Vip3 was proposed to form pores in host cell membranes after activation by proteolysis, which is directly related to their toxicity (23, 24, 34). Our work found that, in addition to the pore-forming ability post proteolytic activation, DI also plays a critical role in maintaining the stability of the Vip3Aa in the presence of midgut proteases and prevent it from excessive hydrolysis by maintaining the proper tetramerization of the Vip3Aa protoxin. Additionally, structural analysis of the Vip3 protoxin demonstrated that in addition to DI, DII and DIII are also involved in the tetramerization of Vip3 (34, 36). *Via* alanine scanning, Banyuls *et al.* (45) found a series of amino acid mutations—some located on the tetrameric interface of DI-DIII—that could significantly decrease the insecticidal activity of Vip3Af. Some mutations in them, such as P171A and F229A, have also been found to affect the tetramerization of Vip3Af (45) and the stability of Vip3Af to trypsin (30), further indicating that proper tetramerization is required for Vip3Aa’s stability in the protease-enriched host insect midgut.

In previous works, DIII was shown to play a major role in the binding between Vip3Aa and Sf9 cells (35), and DI-DIII of Vip3Af was found to specifically bind to Sf21 cells (37). Our fluorescence-based cell-binding assays showed that the ability of DII-DIII to bind cultured Sf9 cells and the midgut epithelium of *S. frugiperda* was similar to that of the full-length Vip3Aa protein. Truncation variants of Vip3Aa lacking DII and DIII, or containing only one of these domains, could not bind to target cells, indicating that DII and DIII together are the receptor-binding domains of Vip3. In addition, as shown in Figure 1*C*, although some proteins are internalized into Sf9 cells, it is clear that Vip3Aa and DII-DIII mainly bind to the cell membrane of Sf9 cells. However, for the frozen tissue sections, we found that in addition to binding to the cell membrane, many Vip3Aa proteins are also located in midgut epithelial cells. Because frozen midgut epithelium sections are not living cells, they do not have innate internalization functions. In addition to the inadequate resolution at the time of image acquisition, we think that the possible reasons for these observations are the effects on epithelial cell permeability during frozen section preparation and the diffusion of Vip3Aa’s binding epitopes in cells during the long fluorescent staining process. Furthermore, several studies have found that Cry toxins can be internalized into midgut cells after ingestion (46, 47). Whether Vip3Aa can enter into midgut cells and functions intracellularly need further study.

We also found that DI-DIII, like full-length Vip3Aa, could be activated by trypsin or MJ proteolysis and maintain the tetrameric architecture. The activated DI-DIII retains significant toxicity against cultured Sf9 cells, similar to activated full-length Vip3Aa. Additionally, Quan *et al.* (37) also found the truncated DI-DIII of Vip3Af, obtained from trypsin-treated Vip3Af^W552A^, to be as toxic to cultured Sf21 cells as the full-length Vip3Af. Our study together with previous report further demonstrated that DI-DIII is the functional and toxic core of Vip3 proteins that is active against insect cell lines.

Structural studies on Vip3 have revealed that DV contains a glycan-binding motif (33, 34, 35). However, the specific functions of Vip3 DV remain unclear. We found that DV is not involved in binding of midgut epithelium cells and the cytotoxicity of Vip3Aa, however, the insecticidal toxicity of Vip3Aa-lacking DV was lost completely. Previous studies also showed that several mutations in DV dramatically reduced the toxicity of Vip3Af (45). These results indicate that DV is essential to the insecticidal activity of Vip3Aa *in vivo*. Structural analysis of DV revealed that it shares high structural similarity to the family 16 carbohydrate-binding module of S-Layer–associated multidomain endoglucanase (RCSB ID 2ZEY) (35), which is a carbohydrate-binding domain with high specificity to β-1,4-glucose or β-1,4-mannose polymers (48). This structural similarity implied that DV might be a glycan-binding domain. However, which part of the insect midgut and what kind of sugars DV binds to were undetermined. In this work, we unexpectedly found that Vip3Aa could bind to the PMa *via* the glycan-binding activity of DV. Several amino acid residue mutations in the predicted glycan-binding pocket of DV significantly reduced the PMa-binding ability and insecticidal activity of Vip3Aa. Together, these findings indicate that DV contributes to the Vip3Aa’s toxic processes inside the host insect midgut and functions by binding to the glycans of the PMa. Therefore, we propose that DV of Vip3Aa is a PMa-binding domain. Furthermore, previous works also proposed that DV may have other potential functions in the toxicity of Vip3A, such as participating in sustaining the conformational change necessary for Vip3Aa’s toxicity (49) and affecting the stability of Vip3Af in the proteolytic activation process (45). This implies that DV may play multiple roles in the insecticidal process of Vip3Aa, which needs further clarification.

Additionally, the main structural components of the PMa are chitin and its derivatives, which is a homopolymer of β-1,4-N-acetyl-D-glucosamine or its derivative D-glucosamines (42, 43). MST tests showed that Vip3Aa could bind to chitotriose with relatively low-binding affinity, close to millimolar ranges, which is typical of most toxin–glycan interactions (50, 51, 52). However, when multiple copies of the glycan receptors are displayed on the surface, mimicking how they exist on the cell membrane, the binding affinity between toxin and glycan can be increased significantly through multivalent interactions (53, 54, 55). Another explanation for this is that among the complicated glycan components of the PMa, the chitotriose we used may not the best target for Vip3Aa. Additionally, the low-binding affinity may be conducive to the ability of Vip3Aa to separate from the PMa and further target receptors on the surface of midgut epithelial cells.

Moreover, Cry1Ac has also been reported to bind to the PMa, but this is thought to be a resistance mechanism by insects to prevent Cry protein from further binding to midgut tissues (40, 41, 56). In contrast, we hypothesized that the interaction between Vip3Aa and the PMa might facilitate Vip3Aa exert toxicity to *S. frugiperda*. Further studies are required to define processes required for insecticidal toxicity after binding of Vip3Aa to the PMa by its DV. Similar to DV, DIV is also required for the insecticidal activity of Vip3Aa *in vivo* (37), but its specific function is still unclear. Although, DIV was also presumed to contain a glycan-binding motif, the size and charge distribution of the glycan-binding pockets between DIV and DV are quite different (35). In this work, we found that Vip3Aa’s binding to the midgut tissue or PMa is independent of DIV (Figs. 1*E* and 4*B*), which further implies that DIV may have different functions from DV. The proteolysis assays also showed that DIV is resistant to the digestion by MJ (Fig. S8*A*). Therefore, the biological function of DIV in the toxic process of Vip3A need further investigation.

It was found that the pH of the lepidopteran larvae midgut lumen is alkaline (57), and further studies showed that there is a pH gradient from midgut lumen (alkaline) to epithelial cells cytoplasm (acidic), and it becomes neutral pH near the midgut epithelial cells (58). In our work, MJ or trypsin proteolysis assays were performed at pH 8.0. As previously reported, proteolysis assays of Vip3A proteins in pH 8.0 and pH 10.0 produced similar digestion patterns (26, 49, 59). Consistent with the previous result, we also found that after digestion, Vip3Aa showed similar digestion patterns on SDS-PAGE under the above two pH conditions (Fig. S8*B*). In addition, the subsequent bioassay results *in vivo* can also well correspond to the proteolysis assay results, indicating that the proteolysis assays at pH 8.0 can largely reflect the proteolytic processing of Vip3Aa in the larval midgut. The effect of pH on the action mechanism of Vip3A has also been studied. The binding of Vip3Aa to the *S. frugiperda* BBMV was reported to be pH dependent, with the binding decreasing as the pH increased in the range from 7.4 to 9.0. pH is also found to affect the membrane insertion and pore-forming activity of the trypsin-activated Vip3Aa; act-Vip3Aa exhibited stronger membrane permeability and pore formation around pH 8.0. These suggested that Vip3A will play a better role in killing target cells when it is close to neutral pH condition. As mentioned above, our cell binding and cytotoxicity assays were also performed in a neutral pH. However, how accurately that our conclusions drawn *in vitro* assays can reflect the processing process of Vip3A proteins in the midgut of larvae and how Vip3A responds to the complex pH changes in the midgut need more in-depth study.

**Figure 6:**
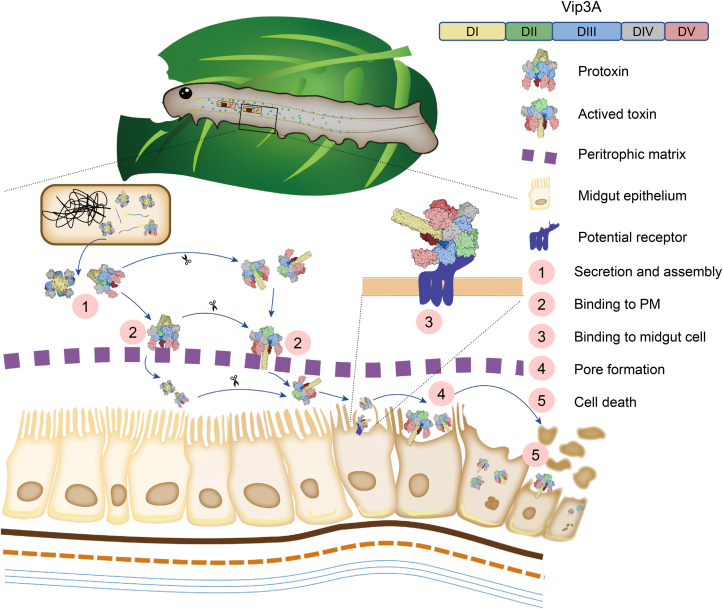
**Proposed primary model of the insecticidal process of Vip3Aa.***1*, Vip3Aa is first secreted and spontaneously assembled into a proper tetramer form to maintain its stability in the complex protease environment of the midgut. *2*, it can then bind to peritrophic matrix through domain V. *3*, following passing the PMa, Vip3Aa will interact with the potential receptors on the midgut epithelium *via* domain II-III. *4*, eventually it will lead to cell death by domain I-III–forming pores on the midgut cells. Since it is not clear in which process Vip3Aa is activated by midgut protease digestion, the possible processes are shown. DI, domain I; DII, domain II; DIII, domain III; PMa, peritrophic matrix; Vip3, vegetative insecticidal protein.

In conclusion, the present study determined the functions of DI, DII-DIII, DI-DIII, and DV of the Vip3Aa protein and their contributions to its toxicity processes against *S. frugiperda* larvae. These results preliminarily reveal part of the insecticidal process of Vip3Aa (Fig. 6). Firstly, Vip3Aa needs to be ingested into the insect midgut in a proper tetramer form to maintain its stability in the complex protease environment of the midgut. It can then bind to PMa through domain V. Following passing the PMa, Vip3Aa would then interact with potential receptors on the midgut epithelium *via* domain II-III. Eventually, it will lead to cell death by domain I-III–forming pores on the midgut cells. However, there are still several issues in the insecticidal process of Vip3A that are not clear, such as in which steps Vip3A is activated by midgut protease digestion, how Vip3A passes through PMa after binding to it *via* DV, and the interaction mechanism between Vip3A and its receptor, which still need further study. By studying the relationship between the functions of the multiple domains and the insecticidal mechanism of Vip3Aa, this study will significantly promote the understanding of the mode of action of Vip3 proteins and facilitate their future rational development and practical application.

## Experimental procedures

### Bacterial strains, cell lines, and insects

*Escherichia coli* B21 (DE3) for plasmid construction and protein purification was cultured at 37 °C in lysogeny broth or agar. Bt 9816C were cultured at 30 °C in lysogeny broth with shaking at 200 rpm. *S. frugiperda* ovarian Sf9 cells were maintained and propagated in Sf-900 II culture medium (Invitrogen) at 27 °C. *S. frugiperda* was used for the bioassays as described below.

### Plasmid construction

The *Vip3Aa* gene from Bt 9816C (60) was cloned into the pET28a vector with an N-terminal 6 × His-small ubiquitin-like motif tag using the Gibson assembly strategy (61, 62). All other truncation variants and point mutations of *Vip3Aa* were generated as full-length *Vip3Aa*. All plasmids were verified by DNA sequencing listed in Table S1.

### Protein expression and purification

The Vip3Aa protein was expressed in *E. coli* BL21(DE3) at 25 °C for 48 h in autoinduction terrific broth medium. Bacterial cells were collected by centrifugation and resuspended in lysis buffer (20 mM Tris–HCl pH 8.0 and 300 mM NaCl). After the cells were lysed by a high-pressure cell crusher (Union-Biotech), the supernatant was collected, run through Ni-NTA agarose resin (Qiagen), and washed with 20 mM Tris–HCl, 300 mM NaCl, 10 mM imidazole, pH 8.0. The small ubiquitin-like motif tag was removed with homemade His-tagged ULPI protease at room temperature (20–30 °C) for 2 h and proteins were then eluted with lysis buffer. The proteins were further purified by HiTrap Q HP ion-exchange chromatography and Superdex 200 Increase 10/300 Gl gel filtration chromatography using an AKTApure chromatography system (GE Healthcare Life Sciences). Proteins corresponding to the molecular weight of the tetramerized Vip3Aa were used for subsequent biochemical and bioassay analyses. The expression and purification steps of other Vip3Aa truncation variants and mutations were similar to those of Vip3Aa. The concentration of proteins was quantified by a NanoPhotometer N60 (Implen). The molar absorption coefficient of each protein was predicted by Protean (DNASTAR) based on the amino acid composition of the protein.

### MJ or trypsin proteolysis assay

Fourth instar larvae of *S. frugiperda* were anesthetized on ice and then dissected to collect midguts. PMa containing the food bolus were removed, and the midgut tissues were mixed and centrifuged at 4 °C for 10 min at 16,000*g*. The supernatant was distributed in small aliquots and stored at −80 °C.

Vip3Aa protein (15 μg) was incubated with MJ at a ratio of 1:16 (MJ: Vip3Aa, wt:wt) in a final volume of 40 μl and incubated at 27 °C for 12 h. Prior to electrophoresis, 1 mM 4-(2-aminoethyl)-benzenesulfonyl fluoride protease inhibitor was used to stop the reaction, followed by SDS-PAGE loading buffer, and then the samples were boiled for 5 min and analyzed by 15% SDS-PAGE.

For trypsin proteolysis assay, Vip3Aa protein (15 μg) was incubated with trypsin at a ratio of 1:50 (Trypsin: Vip3Aa, wt: wt), at 4 °C for 6 h. To purify the proteolysis activated protein, the reaction sample was subjected to size-exclusion chromatography in a Superdex 200 Increase 10/300 Gl column using an AKTApure chromatography system. For electrophoresis, the samples were processed and analyzed by SDS-PAGE as indicated above.

### Dynamic light scattering

DLS measurements were performed using a DynaPro NanoStar instrument (Wyatt Technology) equipped with a 658-nm laser and a 90° back-scattering detector. After centrifugation at 16,000*g* for 10 min at 4 °C, protein samples (0.5 mg/ml) were measured in DLS experiments at 25 °C, to obtain the average hydrodynamic size distribution. Each sample was recorded in triplicate using at least eight data sets acquired for 10 s each. The correlation function was analyzed using the general-purpose method in the software provided by the supplier (Wyatt Technology). Average values of replicates (n = 3) are reported.

### PTS assay

The PTS assays were performed as described by Zhong *et al.* (63). Briefly, the purified Vip3Aa protein and its mutants were dissolved in analysis buffer (20 mM Tris–HCl, 300 mM NaCl, pH = 8.0) to reach the final concentration of 0.5 mg/ml and tested using a protein thermal shift dye kit (Thermo Fisher Scientific) according to the manufacturer’s instructions. The reaction mixture (20 μl) was made and used in an Applied Biosystems Quant Studio 3 Real-Time PCR System (Applied Biosystems Inc). The temperature was increased continuously from 25 °C to 99 °C, at 1.6 °C/s with the following settings for excitation filter: ×4 (580 ± 10 nm) and emission filter: m4 (623 ± 14 nm). Melting curve data were analyzed by protein thermal shift software V1.4 (https://www.thermofisher.cn/order/catalog/product/4466037) (Applied Biosystems Inc) and derivative Tm values were calculated.

### MST assay

MST assays to determine the binding of Vip3Aa proteins and chitotriose were performed as described by Jiang *et al.* (19) with some modifications. Briefly, the purified Vip3Aa-GFP (a fusion protein of Vip3Aa and GFP) and its variants were kept constant at 50 nM in buffer (20 mM Tris (pH 8.0), 300 mM NaCl, and 0.05% (v/v) Tween-20), and chitotriose was titrated from 0.61 μM to 20 mM. Samples were incubated for 20 min at room temperature (20–30 °C) and then loaded into standard, treated capillaries, and analyzed with a NanoTemper Monolith NT.115 (NanoTemper Technologies) at 25 °C. The laser power was set to 20% and the LED power was set to 40%. Normalization of the fluorescence signal and fitting to the Hill equation were performed using the software MO Affinity Analysis v2.3 (https://shop.nanotempertech.com/en/moaffinity-analysis-software-1-license-30) (NanoTemper Technologies). For each sample, the whole procedure was performed three times to yield independent triplicates.

### Cryosectioning of midgut tissue

Fourth instar larvae of *S. frugiperda* were anesthetized on ice for 10 min and then dissected to expose midguts. The midgut tissues were extracted and washed twice in cold PBS buffer (3.2 mM Na_2_HPO_4_, 0.5 mM KH_2_PO_4_, 1.3 mM KCl, 135 mM NaCl, pH 7.4) after the PMa with food bolus removal, followed by fixing in ice-cold fresh 4% paraformaldehyde for 5 h at 4 °C. After washing three times with PBS, the tissues were dehydrated with 15% and 30% sucrose solution overnight for cryoprotection. The midguts were embedded in tissue Tek OCT compound overnight and subjected to cryosectioning to generate 8-μm thick sections. Sections were transferred to slides and stored at −80 °C until use.

### PMa extraction

The fourth instar larvae of *S. frugiperda* were anesthetized on ice for 10 min and then dissected to expose midguts. The midguts were torn apart with tweezers, the PMas with the food bolus were pull out, and then fixed in fresh, ice-cold 4% paraformaldehyde for 1 h. The fixed PMas were dissected with a blade, then thoroughly cleaned in ice-cold PBS buffer three times to remove food residues, and finally stored at 4 °C for later use.

### BBMV preparation and binding assays

The BBMV preparation were performed as described by Wolfersberger *et al.* (64). In brief, midguts were dissected from fifth instar larvae of *S. frugiperda*, BBMV were obtained from the dissected midguts following the differential magnesium precipitation method. Proteins in the purified BBMV were determined by Bradford protein assay (65). BBMV were frozen in liquid nitrogen and stored at −80 °C until used.

The BBMV were centrifuged for 5 min at 16,000*g* and resuspended in binding buffer (PBS buffer, 0.5% bovine serum albumin (BSA), pH 7.4). Two micrograms of Vip3Aa proteins were incubated with 20 μg of BBMV at 4 °C for 2h. The BBMV were recovered by centrifuging at 16,100*g* for 10 min at 4 °C, the supernatant was removed and the pellet was washed three times with ice-cold binding buffer. The final BBMV pellets were resuspended in 10 μl of PBS buffer containing 1 mM 4-(2-aminoethyl)-benzenesulfonyl fluoride. Then, the samples were separated by 15% SDS-PAGE and analyzed by Western blotting with polyclonal rabbit anti-Vip3Aa antibody as described in previous work (19).

### Immunofluorescent staining and confocal microscopy

Vip3Aa protein and its truncation variants were fluorescently labeled using a Cy3-SE fluorescent dye (Solarbio) following the vendor’s recommendation. Cy3-SE dye has a succinimidyl ester moiety that reacts with primary amines of proteins to form stable dye–protein conjugates. Purified protein preparations (0.5 mg/ml) were incubated with reactive dye in a ratio of 1:10 for 30 min at room temperature (20–30 °C) and further purified using Superdex 200 Increase 10/300 Gl gel filtration chromatography to separate the dye–protein conjugates from free dye.

Sf9 cells with a density of 5 × 10^4^ cells per ml were seeded into laser confocal culture dishes. After overnight culture, the cells were treated with Cy3-labeled Vip3Aa or its truncations (0.1 μM) for 6 h. After treatment, cells were washed three times with PBS to remove unbound ligands and fixed with freshly prepared 4% paraformaldehyde at 37 °C for 20 min. Cellular cortical actin and nuclei were labeled for 30 min with FITC-phalloidin (2 μg/ml) (Sigma Aldrich) and 4′,6-diamidino-2-phenylindole (0.5 μg/ml) (Sigma). Cell images were captured using a Zeiss LSM 900 laser confocal microscope.

The frozen tissue sections were washed with PBS twice and blocked in 3% BSA/PBS for 2 h at 4 °C. The slides were incubated with Cy3-labeled proteins (0.1 μM) overnight at 4 °C. Then the sections were washed with PBS and the nuclei were counterstained with 4′,6-diamidino-2-phenylindole (0.5 μg/ml) for 30 min. The slides were mounted in antifade mounting solution (Beyotime). Digital photomicrographs were taken using a Zeiss LSM 900 laser confocal microscope. For the specific binding assays, slides were incubated with Cy3-labeled DII-DIII (0.1 μM) and an excess of unlabeled proteins (40 μM) at the same time, and fluorescence images were obtained as indicated above.

The PMas were blocked in 1% BSA/PBS for 1 h at 4 °C, then incubated with Cy3-labeled proteins (0.05 μM) overnight at 4 °C. PMas were then stained with FITC-conjugated wheat germ agglutinin (10 μg/ml) for 15 min at room temperature (20–30 °C). After washing four times with PBS, the PMas were transferred to slides and mounted in antifade mounting solution (Beyotime). Digital photomicrographs were taken using a Zeiss LSM 900 laser confocal microscope. For the competitive binding assays, the PMas were incubated with Cy3-labeled proteins (0.05 μM) and 1 mM chitotriose at the same time, and fluorescence images were obtained as indicated above.

### Cytotoxicity assays

A total of 100 μl of cells with a density of 5 × 10^4^ cells per ml were seeded into 96-well culture plates separately. After 4 h of incubation, the cells were treated with different Vip3Aa proteins (100 μg/ml) for 72 h. Cell images were captured using a Nikon TI-E inverted Microscope. Cell counting kit-8 (Med Chem Express) reagent was then added to each well. After incubating at 28 °C for 1.5 h, the absorbance was measured in a microplate reader (Tecan) at 450 nm. Treatment with sterile protein buffer and trypsin (2 μg/ml) was used as a control. All tests were performed in triplicate and were repeated at least three times. Cell viability (%) = average absorbance of treated group/average absorbance of control group × 100%.

### Bioassay

Briefly, the assays were assessed using a surface contamination method with *S. frugiperda* larvae, which were maintained in a rearing chamber at 27 °C, with 50% relative humidity and a 16:8 h light:dark photoperiod. The artificial diet was poured in a 24-well plate (about 5 mm thick per hole). Different concentrations of Vip3Aa proteins were spread on the diet. Tris buffer (20 mM Tris–HCl, 300 mM NaCl, pH 8.0) was used as a blank control. After the liquid was completely dry, 24 larvae of *S. frugiperda* were tested at each concentration. Three independent replicates were conducted and mortality was recorded after 5 days. The larvae remaining in the initial instar stage were considered as functional mortality and were also recorded in the number of dead larvae. The lethal concentration values were calculated by GraphPad Prism v.8.0 (https://www.graphpad.com/) (GraphPad).

### Molecular docking analysis

LeDock (http://www.lephar.com/index.htm) was used to dock small molecules into the Vip3Aa structure. The original protein structure (PDB: 6VLS) used for docking is from our previous work (35). The carbohydrate structures of acetyl chitotriose (PubChem CID: 123774) and chitotriose (PubChem CID: 121978) were downloaded from PubChem. Before docking simulation, the protein was preprocessed with LeDock software. Taking the carbohydrate-binding module16 structure (PDB: 3OEA) as a reference, the simulation box was fixed at the possible binding sites, and the size of the box was set to 15 Å × 15 Å × 25 Å in all three dimensions. The calculation yielded 20 possible models, of which the one showing better fitting to the potential glycan-binding pocket was selected to present possible interactions between the glycan and protein.

### Statistical analysis

At least three biological replicates were performed for all experiments. Data are shown as arithmetic mean ± SD. All statistical data were calculated using GraphPad Prism v.8.0 (Prism; GraphPad Software, Inc). For comparisons of the means of two groups, unpaired two-sided *t*-tests were used. For comparisons of multiple groups with a control group, one-way ANOVA was used. The significance of mean comparison was annotated as follows: not significant; ∗*p* < 0.05, ∗∗*p* < 0.01, ∗∗∗*p* < 0.001. *p* < 0.05 was considered to be statistically significant.

## Data availability

All data are contained within the article.

## Supporting information

This article contains supporting information.

## Conflict of interest

The authors declare that they have no conflicts of interest with the contents of this article.
