# Supplementary material for: Functional characterization of Vip3Aa from Bacillus thuringiensis reveals the contributions of specific domains to its insecticidal activity
Source: J Biol Chem. 2023 Feb 9;299(3):103000. doi: 10.1016/j.jbc.2023.103000 (PMC10017365; doi:10.1016/j.jbc.2023.103000)
Supplement: Supplemental Figures S1–S8 and Table S1 [file mmc1.pdf]

Supporting Information

Functional characterization of Vip3Aa from *Bacillus thuringiensis* reveals the contributions of specific domains to its insecticidal activity

Kun Jiang[1, ‡], Zhe Chen[1, ‡], Yuanrong Zang[1], Yiting Shi[2,3], Chengbin Shang[2], Xuyao Jiao[1], Jun Cai[4], Xiang Gao[1,*]

[1] State Key Laboratory of Microbial Technology, Shandong University, Qingdao 266237, China

[2] School of Life Sciences, Shandong University, Qingdao 266237, China

[3] Taishan College, Shandong University, Jinan 250100, China.

[4] Department of Microbiology, College of Life Sciences, Nankai University, Tianjin 300071, China

‡ These authors contributed equally to this work.

*Correspondence: Xiang Gao, xgao@email.sdu.edu.cn

This file contains：

Figures S1 – S8

Table S1

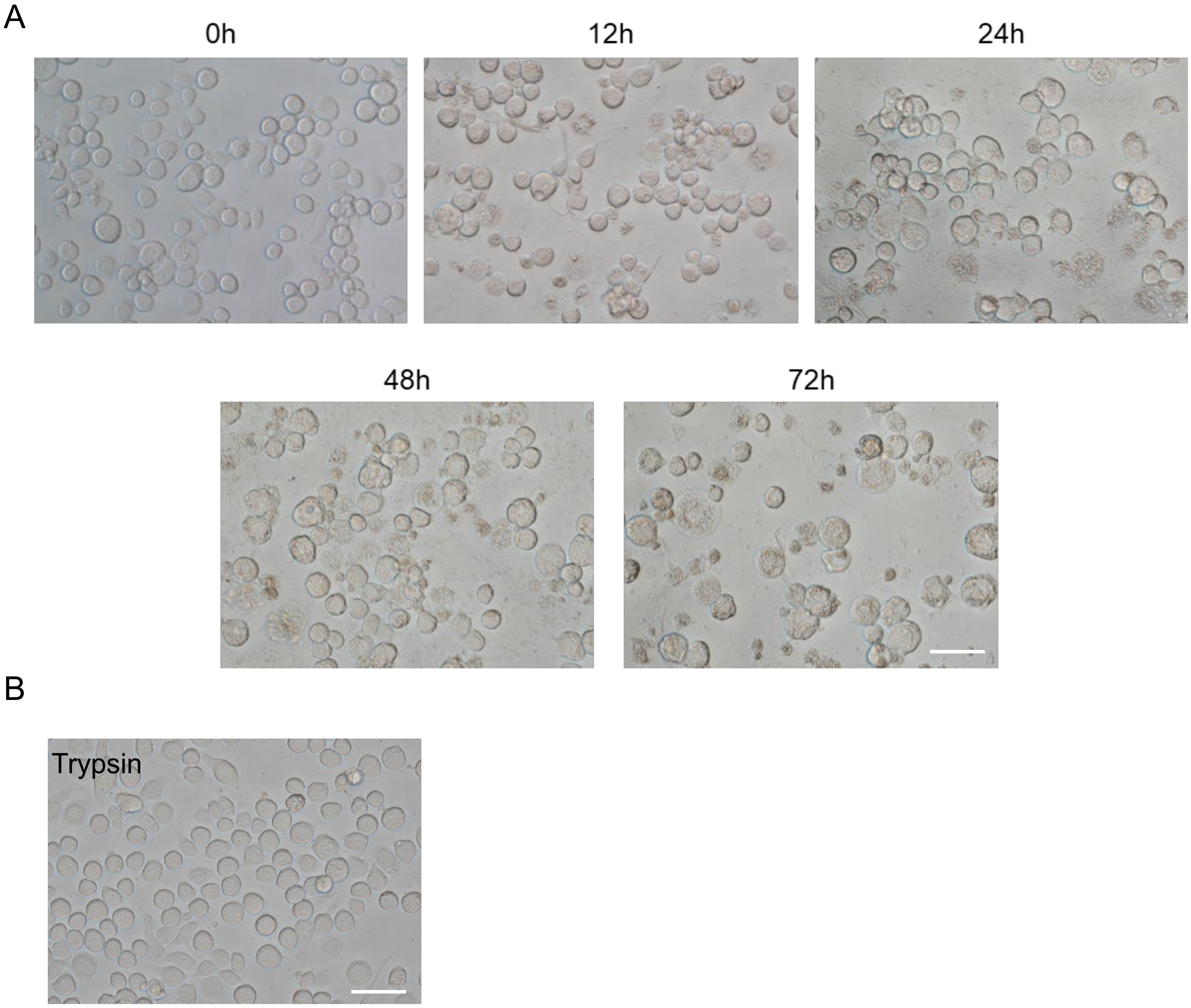

Figure S1. Morphology of Sf9 cells exposed to trypsin-activated Vip3Aa and the indicated Vip3Aa mutants.

(A) Microscopic views of Sf9 cells treated with trypsin-activated DI-DIII for 12, 24, 48, and 72 hours. Scale bar, 20 μm.

(B) Microscopic views of Sf9 cells treated with trypsin (2μg/ml) for 72 hours. Scale bar, 20 μm.

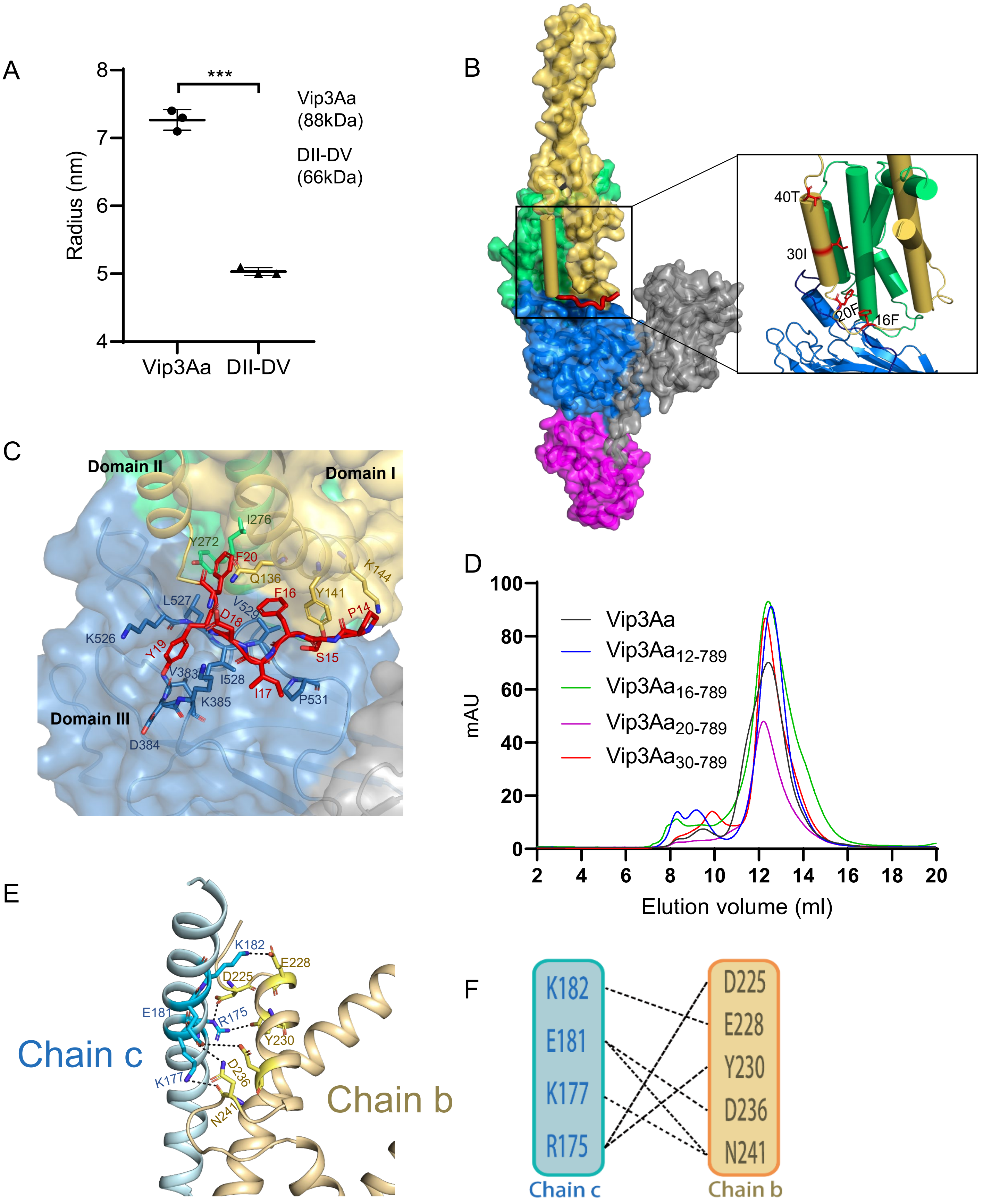
A
Radius (nm)
8
7
6
5
4
***
Vip3Aa
(88kDa)
DII-DV
(66kDa)
Vip3Aa
DII-DV
B
40T
30I
20F
16F
C
Domain II
Domain I
Domain III
I276
Y272
F20
Q136
K144
F16
Y141
L527
V529
P14
D18
K526
Y19
S15
V383
I528
I17
P531
K385
D384
D
100
80
60
40
20
0
mAU
Vip3Aa
Vip3Aa12-789
Vip3Aa16-789
Vip3Aa20-789
Vip3Aa30-789
2
4
6
8
10
12
14
16
18
20
Elution volume (ml)
E
K182
E228
D225
E181
R175
Y230
K177
D236
N241
Chain c
Chain b
F
K182
E181
K177
R175
D225
E228
Y230
D236
N241
Chain c
Chain b

Figure S2. Domain I is involved in the tetramerization of Vip3Aa protoxin.

(A) Dynamic light scattering analysis of Vip3Aa and DII-DV (0.5 mg/ml). Data are expressed as the mean ±SD from three independent experiments; ***, $P < 0.001$ by unpaired two-tailed Student's *t*-tests.

(B) Structure showing the N-terminal end of Vip3Aa protoxin and its interaction with domain I and domain III (PDB 6tfj).

(C) The ribbon highlights the interactions between the N-terminal amino acids (14-22) (red) of Vip3Aa protoxin and its domain I (yellow) and domain III (blue); the interacting residues are shown as sticks.

(D) Size-exclusion chromatography analysis of the purified Vip3Aa, $Vip3Aa_{12\text{-}789}$, $Vip3Aa_{16\text{-}789}$, $Vip3Aa_{20\text{-}789}$, and $Vip3Aa_{30\text{-}789}$ for subsequent assays. The samples were loaded on a Superdex 200 Increase 10/300 GL column.

(E) The dotted lines indicate the interactions of the amino acids R175, K177, E181, and K182 of chain c and chain b, corresponding to the tetrameric Vip3Aa protoxin (PDB 6tfj); the interacting residues are shown as sticks.

(F) Close up view of the polar interaction of (E). The K177 and K182 in the Chain c interact with N241 and E228 in the Chain b respectively. The R175 in the Chain c interact with D225 and Y230 in the Chain b, and the E181 in the Chain c interact with D236 and N241 in the Chain b.

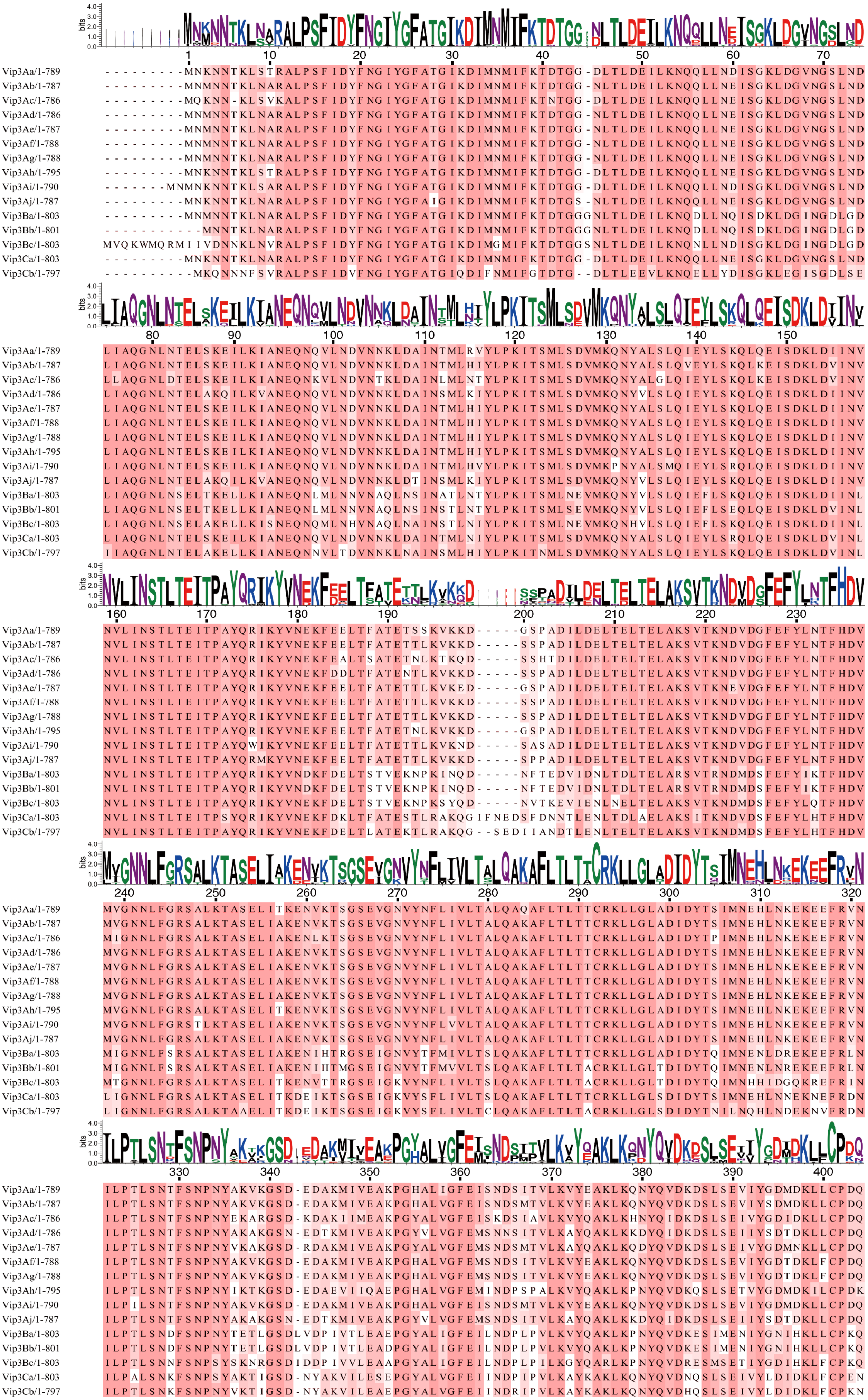

Vip3Aa/1-789 ---------MNKNNTKLSTRALPSFIDYFNGIYGFATGIKDIMNMIFKTDTGG-DLTLDEILKNQQLLNDISGKLDGVNGSLND
Vip3Ab/1-787 ---------MNMNNTKLNARALPSFIDYFNGIYGFATGIKDIMNMIFKTDTGG-NLTLDEILKNQQLLNEISGKLDGVNGSLND
Vip3Ac/1-786 ---------MQKNN-KLSVKALPSFIDYFNGIYGFATGIKDIMNMIFKTNTGG-DLTLDEILKNQQLLNEISGKLDGVNGSLND
Vip3Ad/1-786 ---------MNMNNTKLNARALPSFIDYFNGIYGFATGIKDIMNMIFKTDTGG-NLTLDEILKNQQLLNEISGKLDGVNGSLND
Vip3Ae/1-787 ---------MNMNNTKLNARALPSFIDYFNGIYGFATGIKDIMNMIFKTDTGG-NLTLDEILKNQQLLNEISGKLDGVNGSLND
Vip3Af/1-788 ---------MNMNNTKLNARALPSFIDYFNGIYGFATGIKDIMNMIFKTDTGG-NLTLDEILKNQQLLNEISGKLDGVNGSLND
Vip3Ag/1-788 ---------MNMNNTKLNARALPSFIDYFNGIYGFATGIKDIMNMIFKTDTGG-NLTLDEILKNQQLLNEISGKLDGVNGSLND
Vip3Ah/1-795 ---------MNKNNTKLSTRALPSFIDYFNGIYGFATGIKDIMNMIFKTDTGG-DLTLDEILKNQQLLNEISGKLDGVNGSLND
Vip3Ai/1-790 -------MNMNKNNTKLSARALPSFIDYFNGIYGFATGIKDIMNMIFKTDTGG-DLTLDEILKNQQLLNDISGKLDGVNGSLND
Vip3Aj/1-787 ---------MNKNNTKLNARALPSFIDYFNGIYGFAIGIKDIMNMIFKTDTGS-NLTLDEILKNQQLLNEISGKLDGVNGSLND
Vip3Ba/1-803 ---------MNMNNTKLNARALPSFIDYFNGIYGFATGIKDIMNMIFKTDTGGGNLTLDEILKNQDLLNQISDKLDGINGDLGD
Vip3Bb/1-801 ----------MNNTKLNARALPSFIDYFNGIYGFATGIKDIMNMIFKTDTGGGNLTLDEILKNQDLLNQISDKLDGINGDLGD
Vip3Bc/1-803 MVQKWMQRMIIVDNNKLNVRALPSFIDYFNGIYGFATGIKDIMGMIFKTDTGGSNLTLDEILKNQNLLNDISGKLDGINGDLGD
Vip3Ca/1-803 ---------MNKNNTKLNARALPSFIDYFNGIYGFATGIKDIMNMIFKTDTGG-DLTLDEILKNQQLLNEISGKLDGVNGSLND
Vip3Cb/1-797 ---------MKQNNNFSVRALPSFIDVFNGIYGFATGIQDIFNMIFGTDTG--DLTLEEVLKNQELLYDISGKLEGISGDLSE

Vip3Aa/1-789 LIAQGNLNTELSKEILKIANEQNQVLNDVNNKLDAINTMLRVYLPKITSMLSDVMKQNYALSLQIEYLSKQLQEISDKLDIINV
Vip3Ab/1-787 LIAQGNLNTELSKEILKIANEQNQVLNDVNNKLDAINTMLHIYLPKITSMLSDVMKQNYALSLQVEYLSKQLKEISDKLDVINV
Vip3Ac/1-786 LLAQGNLDTELSKEILKIANEQNKVLNDVNTKLDAINLMLKIYLPKITSMLSDVMKQNYALGLQIEYLSKQLKEISDKLDVINV
Vip3Ad/1-786 LIAQGNLNTELAKQILKVANEQNQVLNDVNNKLDAINSMLKIYLPKITSMLSDVMKQNYVLSLQIEYLSKQLQEISDKLDIINV
Vip3Ae/1-787 LIAQGNLNTELSKEILKIANEQNQVLNDVNNKLDAINTMLHIYLPKITSMLSDVMKQNYALSLQIEYLSKQLQEISDKLDIINV
Vip3Af/1-788 LIAQGNLNTELSKEILKIANEQNQVLNDVNNKLDAINTMLHIYLPKITSMLSDVMKQNYALSLQIEYLSKQLQEISDKLDIINV
Vip3Ag/1-788 LIAQGNLNTELSKEILKIANEQNQVLNDVNNKLDAINTMLHIYLPKITSMLSDVMKQNYALSLQIEYLSKQLQEISDKLDIINV
Vip3Ah/1-795 LIAQGNLNTELSKEILKIANEQNQVLNDVNNKLDAINTMLHIYLPKITSMLSDVMKQNYALSLQIEYLSKQLQEISDKLDIINV
Vip3Ai/1-790 LIAQGNLNTELSKEILKIANEQNQVLNDVNNKLDAINTMLHVYLPKITSMLSDVMKPNYALSMQIEYLSRQLQEISDKLDIINV
Vip3Aj/1-787 LIAQGNLNTELAKQILKVANEQNQVLNDVNNKLDTINSMLKIYLPKITSMLSDVMKQNYVLSLQIEYLSKQLQEISDKLDIINV
Vip3Ba/1-803 LIAQGNLNSELTKELLKIANEQNLMLNNVNAQLNSINATLNTYLPKITSMLNEVMKQNYVLSLQIEFLSKQLQEISDKLDIINL
Vip3Bb/1-801 LIAQGNLNSELTKELLKIANEQNLMLNNVNAQLNSINSTLNTYLPKITSMLSEVMKQNYVLSLQIEFLSEQLQEISDKLDVINL
Vip3Bc/1-803 LIAQGNLNSELAKELLKISNEQNQMLNHVNAQLNAINSTLNIYLPKITSMLNEVMKQNHVLSLQIEFLSKQLQEISDKLDIINL
Vip3Ca/1-803 LIAQGNLNTELSKEILKIANEQNQVLNDVNNKLDAINTMLNIYLPKITSMLSDVMKQNYALSLQIEYLSRQLQEISDKLDVINL
Vip3Cb/1-797 IIAQGNLNTELAKELLKIANEQNNVLTDVNNKLNAINSMLHIYLPKITNMLSDVMKQNYALSLQIEYLSKQLQEISDKLDVINL

Vip3Aa/1-789 NVLINSTLTEITPAYQRIKYVNEKFEELTFATETSSKVKKD-----GSPADILDELTELTELAKSVTKNDVDGFEFYLNTFHDV
Vip3Ab/1-787 NVLINSTLTEITPAYQRIKYVNEKFEELTFATETTLKVKKD-----SSPADILDELTELTELAKSVTKNDVDGFEFYLNTFHDV
Vip3Ac/1-786 NVLINSTLTEITPAYQRIKYVNEKFEALTSATETNLKTKQD-----SSHTDILDELTELTELAKSVTKNDVDGFEFYLNTFHDV
Vip3Ad/1-786 NVLINSTLTEITPAYQRIKYVNEKFDDLTFATENTLKVKKD-----SSPADILDELTELTELAKSVTKNDVDGFEFYLNTFHDV
Vip3Ae/1-787 NVLINSTLTEITPAYQRIKYVNEKFEELTFATETTLKVKED-----GSPADILDELTELTELAKSVTKNEVDGFEFYLNTFHDV
Vip3Af/1-788 NVLINSTLTEITPAYQRIKYVNEKFEELTFATETTLKVKKD-----SSPADILDELTELTELAKSVTKNDVDGFEFYLNTFHDV
Vip3Ag/1-788 NVLINSTLTEITPAYQRIKYVNEKFEELTFATETTLKVKKD-----SSPADILDELTELTELAKSVTKNDVDGFEFYLNTFHDV
Vip3Ah/1-795 NVLINSTLTEITPAYQRIKYVNEKFEELTFATETNLKVKKD-----GSPADILDELTELTELAKSVTKNDVDGFEFYLNTFHDV
Vip3Ai/1-790 NVLINSTLTEITPAYQWIKYVNEKFEELTFATETTLKVKND-----SASADILDELTELTELAKSVTKNDVDGFEFYLNTFHDV
Vip3Aj/1-787 NVLINSTLTEITPAYQRMKYVNEKFEELTFATETTLKVKKD-----SPPADILDELTELTELAKSVTKNDVDGFEFYLNTFHDV
Vip3Ba/1-803 NVLINSTLTEITPAYQRIKYVNDKFDELTSTVEKNPKINQD-----NFTEDVIDNLTDLTELARSVTRNDMDSFEFYIKTFHDV
Vip3Bb/1-801 NVLINSTLTEITPAYQRIKYVNDKFDELTSTVEKNPKINQD-----NFTEDVIDNLTDLTELARSVTRNDMDSFEFYIKTFHDV
Vip3Bc/1-803 NVLINSTLTEITPAYQRIKYVNEKFDELTSTVEKNPKSYQD-----NVTKEVIENLNELTELAKSVTKNDMDSFEFYLQTFHDV
Vip3Ca/1-803 NVLINSTLTEITPSYQRIKYVNEKFDKLTFATESTLRAKQGIFNEDSFDNNTLENLTDLAELAKSITKNDVDSFEFYLHTFHDV
Vip3Cb/1-797 NVLINSTLTEITPAYQRIKYVNEKFDELTLATEKTLRAKQG--SEDIIANDTLENLTELTELAKSVTKNDMDSFEFYLHTFHDV

Vip3Aa/1-789 MVGNNLFGRSALKTASELITKENVKTSGSEVGNVYNFLIVLTALQAQAFLTLTTCRKLLGLADIDYTSIMNEHLNKEKEEFRVN
Vip3Ab/1-787 MVGNNLFGRSALKTASELIAKENVKTSGSEVGNVYNFLIVLTALQAKAFLTLTTCRKLLGLADIDYTSIMNEHLNKEKEEFRVN
Vip3Ac/1-786 MIGNNLFGRSALKTASELIAKENLKTSGSEVGNVYNFLIVLTALQAKAFLTLTTCRKLLGLADIDYTPIMNEHLNKEKEEFRVN
Vip3Ad/1-786 MVGNNLFGRSALKTASELIAKENVKTSGSEVGNVYNFLIVLTALQAKAFLTLTTCRKLLGLADIDYTSIMNEHLNKEKEEFRVN
Vip3Ae/1-787 MVGNNLFGRSALKTASELIAKENVKTSGSEVGNVYNFLIVLTALQAKAFLTLTTCRKLLGLADIDYTSIMNEHLNKEKEEFRVN
Vip3Af/1-788 MVGNNLFGRSALKTASELIAKENVKTSGSEVGNVYNFLIVLTALQAKAFLTLTTCRKLLGLADIDYTSIMNEHLNKEKEEFRVN
Vip3Ag/1-788 MVGNNLFGRSALKTASELIAKENVKTSGSEVGNVYNFLIVLTALQAKAFLTLTTCRKLLGLADIDYTSIMNEHLNKEKEEFRVN
Vip3Ah/1-795 MVGNNLFGRSALKTASELITKENVKTSGSEVGNVYNFLIVLTALQAKAFLTLTTCRKLLGLADIDYTSIMNEHLNKEKEEFRVN
Vip3Ai/1-790 MVGNNLFGRSTLKTASELIAKENVKTSGSEVGNVYNFLVVLTALQAKAFLTLTTCRKLLGLADIDYTSIMNEHLNKEKEEFRVN
Vip3Aj/1-787 MVGNNLFGRSALKTASELIAKENVKTSGSEVGNVYNFLIVLTALQAKAFLTLTTCRKLLGLADIDYTSIMNEHLNKEKEEFRVN
Vip3Ba/1-803 MIGNNLFSRSALKTASELIAKENIHTRGSEIGNVYTFMIVLTSLQAKAFLTLTACRKLLGLTDIDYTQIMNENLDREKEEFRLN
Vip3Bb/1-801 MIGNNLFSRSALKTASELIAKENIHTMGSEIGNVYTFMVVLTSLQAKAFLTLTACRKLLGLTDIDYTQIMNENLNREKEEFRLN
Vip3Bc/1-803 MTGNNLFGRSALKTASELITKENVTTRGSEIGKVYNFLIVLTSLQAKAFLTLTACRKLLGLTDIDYTQIMNHHIDGQKREFRIN
Vip3Ca/1-803 LIGNNLFGRSALKTASELITKDEIKTSGSEIGKVYSFLIVLTSLQAKAFLTLTTCRKLLGLSDIDYTSIMNEHLNNEKNEFRDN
Vip3Cb/1-797 LIGNNLFGRSALKTAAELITKDEIKTSGSEIGKVYSFLIVLTCLQAKAFLTLTACRKLLGLSDIDYTNILNQHLNDEKNVFRDN

Vip3Aa/1-789 ILPTLSNTFSNPNYAKVKGSD-EDAKMIVEAKPGHALIGFEISNDSITVLKVYEAKLKQNYQVDKDSLSEVIYGDMDKLLCPDQ
Vip3Ab/1-787 ILPTLSNTFSNPNYAKVKGSD-EDAKMIVEAKPGHALVGFEISNDSMTVLKVYEAKLKQNYQVDKDSLSEVIYSDMDKLLCPDQ
Vip3Ac/1-786 ILPTLSNTFSNPNYEKARGSD-EDAKIIMEAKPGYALVGFEISKDSIAVLKVYQAKLKHNYQIDKDSLSEIVYGDIDKLLCPDQ
Vip3Ad/1-786 ILPTLSNTFSNPNYAKAKGSN-EDTKMIVEAKPGYVLVGFEMSNNSITVLKAYQAKLKKDYQIDKDSLSEIIYSDTDKLLCPDQ
Vip3Ae/1-787 ILPTLSNTFSNPNYVKAKGSD-RDAKMIVEAKPGYALVGFEMSNDSMTVLKAYQAKLKQDYQVDKDSLSEIVYGDMNKLLCPDQ
Vip3Af/1-788 ILPTLSNTFSNPNYAKVKGSD-EDAKMIVEAKPGHALVGFEMSNDSITVLKVYEAKLKQNYQVDKDSLSEVIYGDTDKLFCPDQ
Vip3Ag/1-788 ILPTLSNTFSNPNYAKVKGSD-EDAKMIVEAKPGHALVGFEMSNDSITVLKVYEAKLKQNYQVDKQSLSEVIYGDTDKLFCPDQ
Vip3Ah/1-795 ILPTLSNTFSNPNYIKTKGSD-EDAEVIIQAEPGHALVGFEMINDPSPALKVYQAKLKPNYQVDKQSLSETVYGDMDKLICPDK
Vip3Ai/1-790 ILPILSNTFSNPNYAKVKGSD-EDAKMIVEAKPGHALVGFEISNDSMTVLKVYEAKLKQNYQVDKDSLSEVIYGDMDKLLCPDQ
Vip3Aj/1-787 ILPTLSNTFSNPNYAKAKGSN-EDTKMIVEAKPGYVLVGFEMSNDSITVLKAYQAKLKKDYQIDKDSLSEIIYSDTDKLLCPDQ
Vip3Ba/1-803 ILPTLSNDFSNPNYTETLGSDLVDPIVTLEAEPGYALIGFEILNDPLPVLKVYQAKLKPNYQVDKESIMENIYGNIHKLLCPKQ
Vip3Bb/1-801 ILPTLSNDFSNPNYTETLGSDLVDPIVTLEADPGYALIGFEILNDPLPVLKVYQAKLKPNYQVDKESIMENIYGNIHKLLCPKQ
Vip3Bc/1-803 ILPTLSNNFSNPSYSKNRGSDIDDPIVVLEAAPGYALIGFEILNDPLPILKGYQARLKPNYQVDRESMSETIYGDIHKLFCPKQ
Vip3Ca/1-803 ILPALSNKFSNPSYAKTIGSD-NYAKVILESEPGYALVGFEIINDPIPVLKAYKAKLKQNYQVDNQSLSEIVYLDIDKLFCPEN
Vip3Cb/1-797 ILPTLSNKFSNPNYVKTIGSD-NYAKVILEAEPGYALVGFEIINDRIPVLKAYKAKLKQNYQVDHQSLSEIVYLDIDKLFCPKN

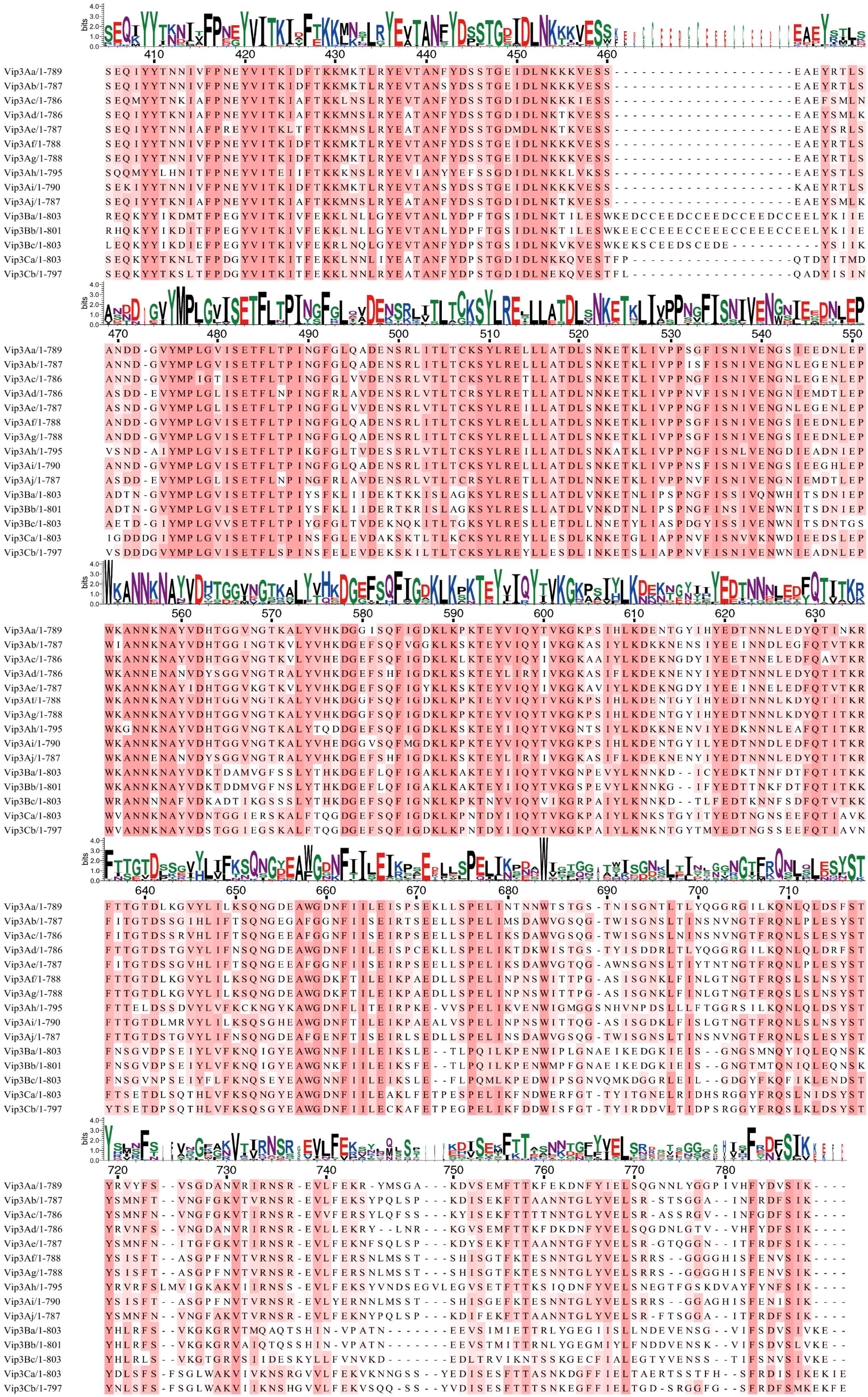

Figure S3. Sequence alignment of the amino acids of Vip3 family proteins.

Sequence alignment of the amino acids of different subclasses of Vip3 family proteins. The Weblogo above indicates frequency and was generated using WebLogo 3: Public Beta.

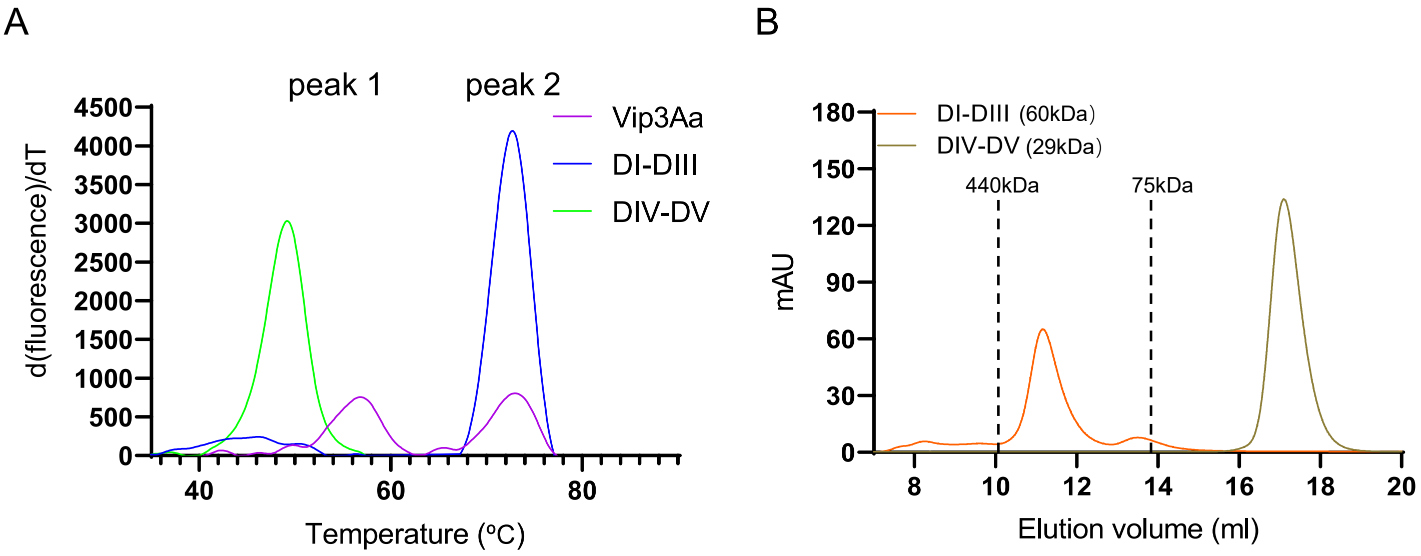

Figure S4. Effects of DI-DIII and DIV-DV on tetramerization of Vip3Aa

(A) Protein thermal shift assay analysis of Vip3Aa, DI-DIII, and DIV-DV (0.5 mg/ml). The thermal shift assays curves are representative of three independent repetitions of each sample.

(B) Size-exclusion chromatography analysis of the purified DI-DIII and DIV-DV for subsequent assays. The samples were loaded on a Superdex 200 Increase 10/300 GL column. The dashed vertical lines indicate the peak positions of the molecular weight standards 75 kDa and 440 kDa.

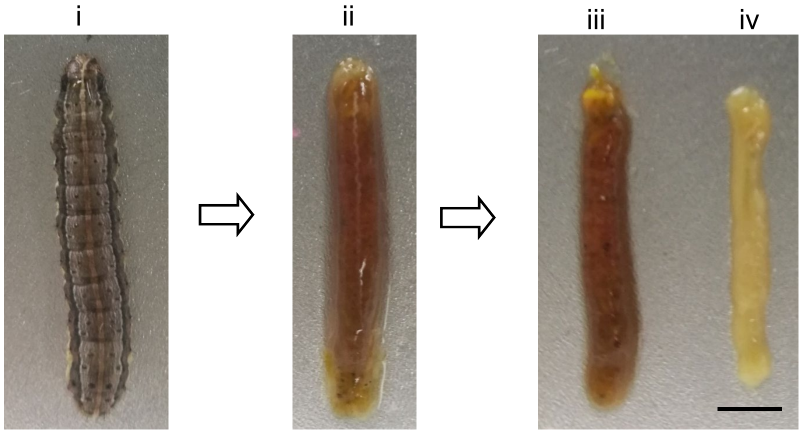

Figure S5. Schematic diagram of anatomy *Spodoptera frugiperda* larvae to obtain PMa.

i : Fourth instar *S. frugiperda* larva, ii : Midgut tissues and its contents after removal of outer epidermis, iii : Peritrophic matrix and its contents, iv : Midgut tissues. Scale bar, 0.5 cm.

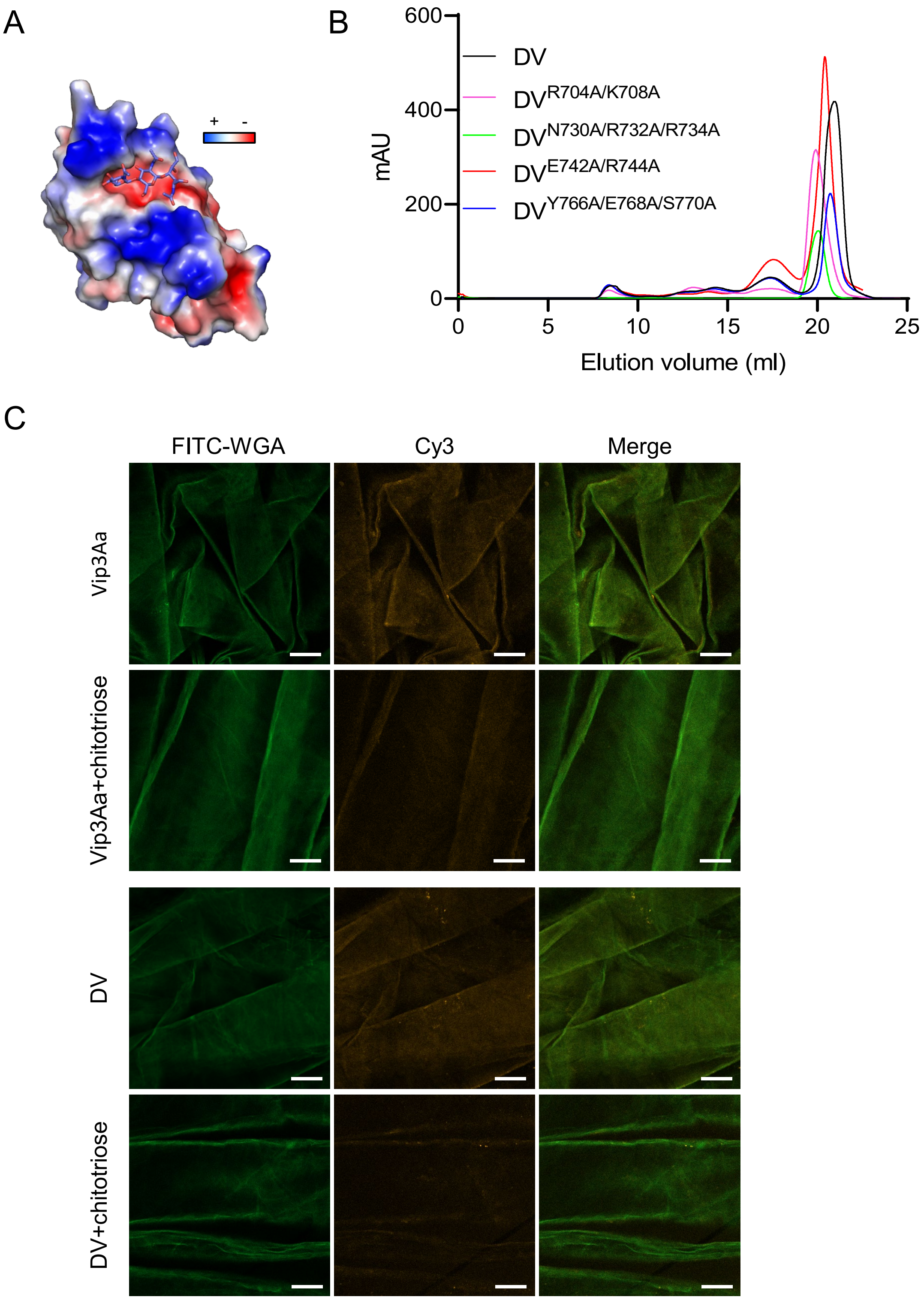
A
+
-
B
600
400
200
0
mAU
DV
DV$^{R704A/K708A}$
DV$^{N730A/R732A/R734A}$
DV$^{E742A/R744A}$
DV$^{Y766A/E768A/S770A}$
0
5
10
15
20
25
Elution volume (ml)
C
FITC-WGA
Cy3
Merge
Vip3Aa
Vip3Aa+chitotriose
DV
DV+chitotriose

Figure S6. Vip3Aa binds to the peritrophic matrix of *S. frugiperda* larvae via Domain V

(A) The surface charge distribution showing the docking of results of the binding between the DV of Vip3Aa (PDB: 6vls) and chitotriose. Blue and red represent positive and negative potentials, respectively. The chitotriose is denoted as a stick (in purple).

(B) Size-exclusion chromatography analysis of the purified DV and the indicated DV mutants for subsequent assays. The samples were loaded on a Superdex 200 Increase 10/300 GL column.

(C) Confocal microscopy images showing the effect of the presence of chitotriose (1 mM) on the binding of Cy3-labeled Vip3Aa and DV (yellow) (0.05 μM) to the PMa of *S. frugiperda* larvae. The PMa is stained with FITC-conjugated wheat germ agglutinin (WGA) (green). Scale bar, 50 μm. The images represent at least three independent experiments.

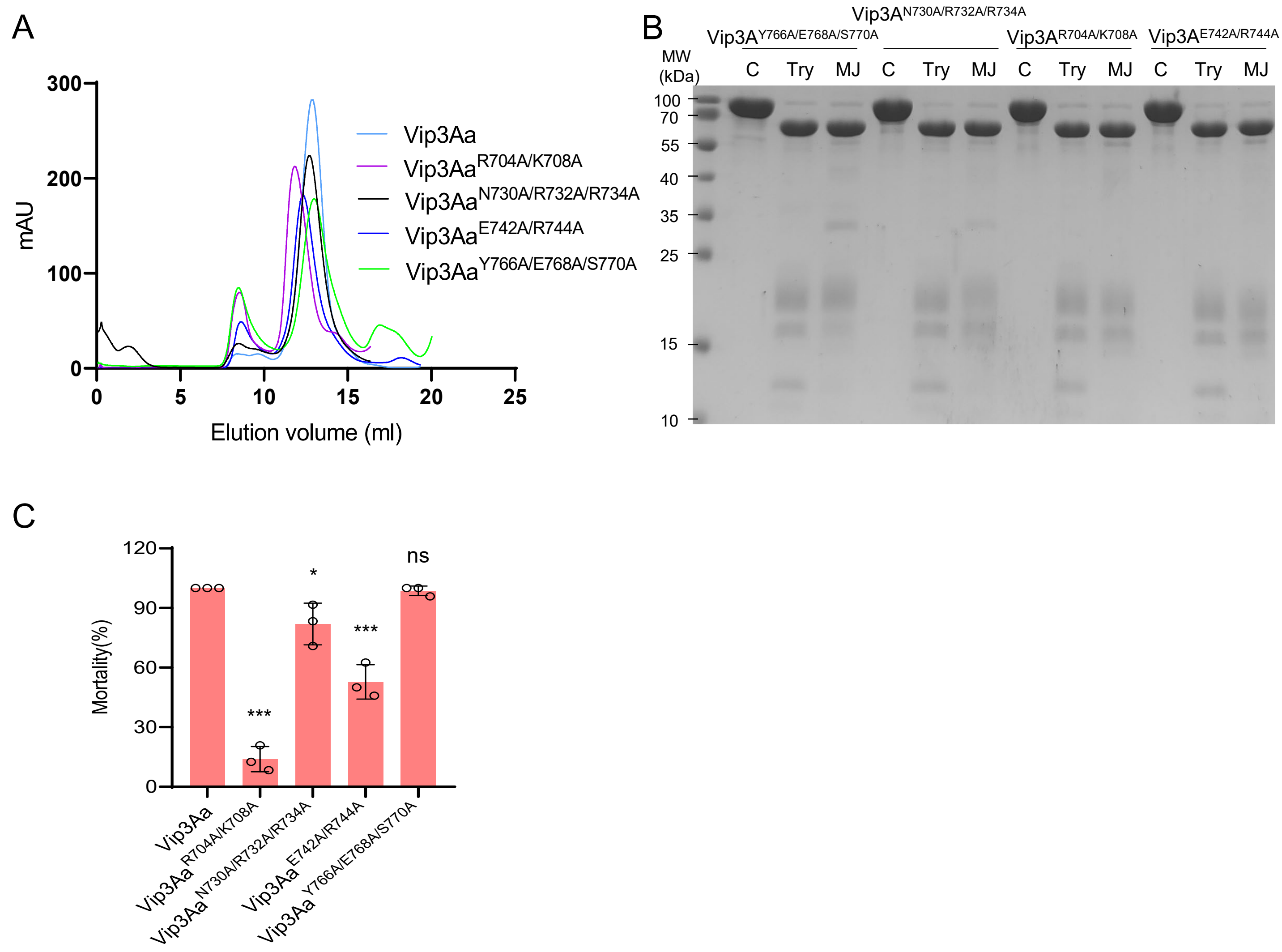

Figure S7. Effects of mutations of Domain V on insecticidal activity of Vip3Aa

(A) Size-exclusion chromatography analyses of the purified Vip3Aa and the indicated mutant proteins. The samples were loaded on a Superdex 200 Increase 10/300 GL column.

(B) SDS-PAGE analysis of the indicated Vip3Aa mutants after treatment with trypsin or *S. frugiperda* MJ. “C”: the proteins untreated as control; “Try”: the proteins treated with trypsin; “MJ”: the proteins treated with MJ.

(C) Insecticidal activity of Vip3Aa and the indicated Vip3Aa mutants against *S. frugiperda* larvae at concentrations of 100 ng/cm^2 (n = 24). Data are expressed as the mean ±SD from three independent experiments. Statistical analysis was performed using one-way ANOVA with Duncan’s MRT; ns, non-significant; *, $P< 0.05$; ***, $P < 0.001$.

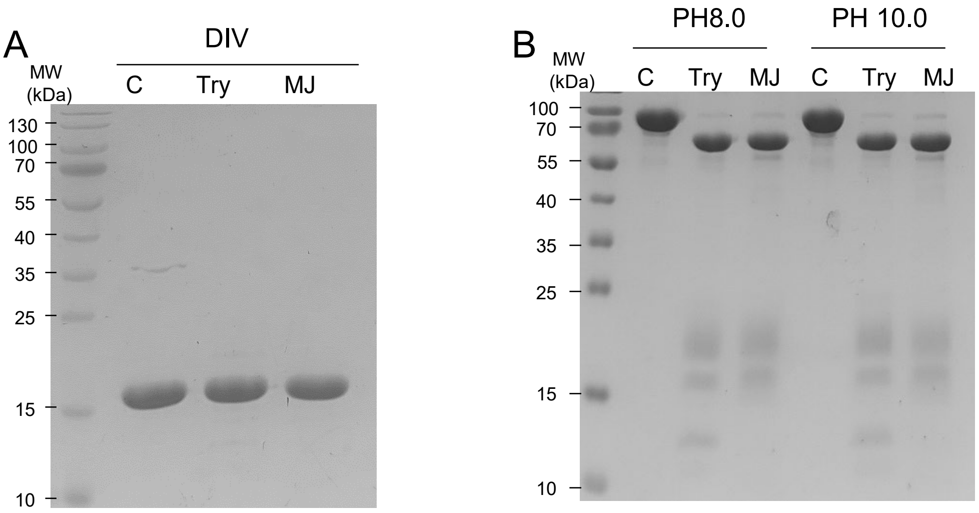

Figure S8. SDS-PAGE analysis of Vip3Aa and DIV after treatment with trypsin or *S. frugiperda* MJ.

(A) SDS-PAGE analysis of DIV after treatment with trypsin or *S. frugiperda* MJ.

(B) SDS-PAGE analysis of Vip3Aa after treatment with trypsin or *S. frugiperda* MJ at PH 8.0 and PH 10.0.

Table S1. Plasmids used in this study.

Plasmid	Source
Plasmid: pET-28b-SUMO-Vip3Aa	This study
Plasmid: pET-28b-SUMO-Vip3Aa-DI-DIV	This study
Plasmid: pET-28b-SUMO-Vip3Aa-DI-DIII	This study
Plasmid: pET-28b-SUMO-Vip3Aa-DI-DII	This study
Plasmid: pET-28b-SUMO-Vip3Aa-DII-DV	This study
Plasmid: pET-28b-SUMO-Vip3Aa-DII-DIII	This study
Plasmid: pET-28b-SUMO-Vip3Aa-DIII-DV	This study
Plasmid: pET-28b-SUMO-Vip3Aa-DIV-DV	This study
Plasmid: pET-28b-SUMO-Vip3Aa-DIII	This study
Plasmid: pET-28b-SUMO-Vip3Aa-DIV	This study
Plasmid: pET-28b-SUMO-Vip3Aa-DV	This study
Plasmid: pET-28b-SUMO-Vip3Aa-12-789	This study
Plasmid: pET-28b-SUMO-Vip3Aa-16-789	This study
Plasmid: pET-28b-SUMO-Vip3Aa-20-789	This study
Plasmid: pET-28b-SUMO-Vip3Aa-30-789	This study
Plasmid: pET-28b-SUMO-Vip3Aa-M4	This study
Plasmid: pET-28b-SUMO-Vip3Aa-DV_R704A/K708A	This study
Plasmid: pET-28b-SUMO-Vip3Aa-DV_N730A/R732A/R734A	This study
Plasmid: pET-28b-SUMO-Vip3Aa-DV_E742A/R744A	This study
Plasmid: pET-28b-SUMO-Vip3Aa-DV_Y766A/E768A/S770A	This study
Plasmid: pET-28b-SUMO-Vip3Aa-GFP-DV	This study
Plasmid: pET-28b-SUMO-Vip3Aa-GFP-DV_R704A/K708A	This study
Plasmid: pET-28b-SUMO-Vip3Aa-GFP-DV_ N730A/R732A/R734A	This study
Plasmid: pET-28b-SUMO-Vip3Aa-GFP-DV_E742A/R744A	This study
Plasmid: pET-28b-SUMO-Vip3Aa-GFP-DV_ Y766A/E768A/S770A	This study
Plasmid: pET-28b-SUMO-Vip3Aa-R704A/K708A	This study
Plasmid: pET-28b-SUMO-Vip3Aa-N730A/R732A/R734A	This study
Plasmid: pET-28b-SUMO-Vip3Aa-E742A/R744A	This study
Plasmid: pET-28b-SUMO-Vip3Aa-Y766A/E768A/S770A	This study
